# BPC 157 as a Therapy for Retinal Ischemia Induced by Retrobulbar Application of L-NAME in Rats

**DOI:** 10.3389/fphar.2021.632295

**Published:** 2021-06-10

**Authors:** Mirna Zlatar, Antonio Kokot, Lovorka Batelja Vuletic, Sanja Masnec, Tamara Kralj, Marija Milkovic Perisa, Ivan Barisic, Bozo Radic, Kristina Milanovic, Domagoj Drmic, Sven Seiwerth, Predrag Sikiric

**Affiliations:** ^1^ Department of Ophthalmology, General Hospital Virovitica, Virovitica, Croatia; ^2^ Department of Anatomy and Neuroscience, Faculty of Medicine Osijek, Josip Juraj Strossmayer University of Osijek, Osijek, Croatia; ^3^ Department of Pathology, School of Medicine, University of Zagreb, Zagreb, Croatia; ^4^ Department of Pharmacology, School of Medicine, University of Zagreb, Zagreb, Croatia

**Keywords:** retina, ischemia, BPC 157, L-NAME, rats

## Abstract

Providing NO-system importance, we suggest that one single application of the NOS-blocker L-NAME may induce retinal ischemia in rats, and that the stable pentadecapeptide BPC 157 may be the therapy, since it may interact with the NO-system and may counteract various adverse effects of L-NAME application. A rat retinal ischemia study was conducted throughout 4 weeks, including fundoscopy, behavior presentation, tonometry, and histology assessment. Retrobulbar L-NAME application (5 mg/kg; 0.5 mg/0.1 ml saline/each eye) in rats immediately produced moderate generalized irregularity in the diameter of blood vessels with moderate atrophy of the optic disc and faint presentation of the choroidal blood vessels, and these lesions rapidly progressed to the severe stage. The specific L-NAME–induced vascular failure points to normal intraocular pressure (except to very transitory increase upon drug retrobulbar administration). When BPC 157 (10 μg; 10 ng/kg, as retrobulbar application, 1 μg; 1 ng/0.1 ml saline/each eye) is given at either 20 min after L-NAME or, lately, at 48 h after L-NAME, the regular retrobulbar L-NAME injection findings disappear. Instead, fundoscopy demonstrated only discrete generalized vessel caliber irregularity with mild atrophy of the optic disc, and then, quite rapidly, normal eye background and choroidal blood vessels, which remain in all of the subsequent periods. Also, histology assessment at 1, 2, and 4 weeks shows that BPC 157 counteracted the damaged inner plexiform layer and inner nuclear layer, and revealed normal retinal thickness. The poor behavioral presentation was also rescued. Thus, while further studies will be done, BPC 157 counteracted L-NAME–induced rat retinal ischemia.

## Introduction

We focused on the stable gastric pentadecapeptide BPC 157 (Sikiric et al., 2014; Sikiric et al., 2018; Sikiric et al., 2020) and retinal ischemia in rats. Reasonably, providing an essential physiological role of the nitric oxide (NO) system (Cudeiro and Rivadulla, 1999; Toda and Nakanishitoda, 2007; Guthrie and Kang-Mieler, 2014), its inhibiting may directly lead to the retinal ischemia and definitively debilitated function.

Thus, we suggest that one single application of the NO-synthase (NOS) blocker N(G)-nitro-L-arginine methyl ester (L-NAME) may induce retinal ischemia in rats, and that the stable pentadecapeptide BPC 157 may be the therapy, since it may interact with the NO-system and may counteract various adverse effects of L-NAME application (see Sikiric et al., 2014). This particular point supports the recent evidence that vasomotor tone is carried out through BPC 157–specific activation of the Src-Caveolin-1-endothelial NOS (eNOS) pathway (Hsieh et al., 1997). Likewise, BPC 157 has a modulatory role (Sikiric et al., 2014) (in particular, to the blood pressure (Sikiric et al., 1997) and thrombocytes function maintenance (Stupnisek et al., 2012,^2015^; Konosic et al., 2019)) and additional interaction with several molecular pathways (Tkalcevic et al., 2007; Chang et al., 2011, 2014; Hsieh et al., 2017; 2020; Kang et al., 2018; Vukojevic et al., 2018; Vukojevic et al., 2020; Park et al., 2020; Wu et al., 2020) [i.e., activation of the VEGFR2-Akt-eNOS signaling pathway without the need of other known ligands or shear stress (Hsieh et al., 2017)]. To illustrate its modulatory role on NO-system functions (Sikiric et al., 2014), BPC 157 may counteract L-NAME hypertension and pro-thrombotic effect as well as L-arginine hypotension and antithrombotic effect (Sikiric et al., 1997; Stupnisek et al., 2015), and induce its own NO-release (Sikiric et al., 1997; Turkovic et al., 2004). Also, BPC 157 may be the free radical scavenger to counteract reperfusion-induced free radical injury (Belosic Halle et al., 2017; Duzel et al., 2017; Luetic et al., 2017; Amic et al., 2018; Drmic et al., 2018; Vukojevic et al., 2018; Sever et al., 2019; Sucic et al., 2019; Kolovrat et al., 2020; Park et al., 2020). In addition, BPC 157, as an agent with cytoprotective effect in the entire gastrointestinal tract (and thereby, innate endothelium protection) (Sikiric et al., 2018; Sikiric et al., 2020), prevents and reverses thrombosis formation (Hrelec et al., 2009; Vukojevic et al., 2018; Gojkovic et al., 2020; Kolovrat et al., 2020). The evidence is provided after abdominal aorta anastomosis (Hrelec et al., 2009) or major vein occlusion (Vukojevic et al., 2018; Gojkovic et al., 2020; Kolovrat et al., 2020). Also, BPC 157 maintains thrombocytes function (Konosic et al., 2019) without interfering with coagulation pathways (Konosic et al., 2019). Finally, when confronted with major vessel occlusion, BPC 157 administration recruits vessels to rapidly activate the collateral pathway (Duzel et al., 2017; Amic et al., 2018; Drmic et al., 2018; Vukojevic et al., 2018; Cesar et al., 2020; Gojkovic et al., 2020; Kolovrat et al., 2020). These responses effectively negate the harmful effect of L-NAME–NOS blockade (Duzel et al., 2017; Amic et al., 2018; Drmic et al., 2018; Cesar et al., 2020). Furthermore, there is evidence that BPC 157 counteracted stroke, given in reperfusion, after clamping of the common carotid arteries (i.e., both early and delayed neural hippocampal damage, and achieving full functional recovery (the Morris water maze test, the inclined beam-walking test, and the lateral push test)) (Vukojevic et al., 2020). Likewise, BPC 157 may ameliorate other peripheral and central neuronal damage (Ilic et al., 2009; Ilic et al., 2010; Ilic et al., 2011a; Ilic et al., 2011b; Gjurasin et al., 2010; Tudor et al., 2010; Klicek et al., 2013; Sikiric et al., 2013; Sikiric et al., 2016; Lojo et al., 2016; Drmic et al., 2017; Medvidovic-Grubisic et al., 2017; Perovic et al., 2019; Wang et al., 2019). This may suggest that BPC 157 administration would counteract retinal disturbances also.

In the previous eye research studies, BPC 157 counteracts atropine-mydriasis, but it also opposes an immediate and hour-lasting miotic effect of L-NAME in rats and guinea pigs, and participates in pupil control potentially *via* NO-mediated and cholinergic mechanisms (Kokot et al., 2016). Likewise, there are the adverse effects of NOS-substrate and L-arginine–induced NO overstimulation, which may be counteracted by the application of BPC 157 as well (Kokot et al., 2016). BPC 157 provided a particular healing and vascular effect since it maintained corneal transparency (Lazic et al., 2005; Masnec et al., 2015). Illustratively, total debridement of corneal epithelium as well as perforating corneal incisions (Masnec et al., 2015) was cured with no corneal neovascularization and no new vessels (Lazic et al., 2005; Masnec et al., 2015).

This may be essential for the effective counteraction of the damaging effect of the retrobulbar L-NAME application. In support, intravenous or intravitreous L-NAME application, given before, would aggravate the harmful effect of the major noxious procedure (i.e., ischemia by increasing intraocular pressure above systolic pressure by the infusion into the anterior chamber; ligating optic nerve) (Imai et al., 1997; Ostwald et al., 1997; Hangai et al., 1999; Sakamoto et al., 2006).

Therefore, by retrobulbar L-NAME (and/or BPC 157 application as therapy), we investigated the degree of ischemia of the retina with fundus images of both eyes, from normal presentation until the strong generalized irregular diameter of blood vessels with severe atrophy of the optic disc, and extremely barely visible (extremely faint presentation) choroidal blood vessels (bright fundus background color). Upon sacrifice, we assessed retinal disturbances, particularly those in the inner nuclear layer and the inner plexiform layer. Likewise, after retrobulbar L-NAME application, we focused on the corresponding animal behavior. The contribution of intraocular pressure elevation in the procedure was also evaluated. BPC 157 was given in the early post–L-NAME time, or later, in a more advanced injury course, at 48 h after L-NAME.

## Materials and Methods

### Animals

Study protocols were conducted in male albino Wistar rats, body weight 200 g, 13 weeks old, in-house bred—animal facility, Department of Pharmacology, School of Medicine, Zagreb, Croatia. Animal facility is registered by the directorate of veterinary; Reg. No: HR-POK-007. Laboratory rats were acclimated for 5 days and randomly assigned to their respective treatment group. Laboratory animals were housed in PC cages in conventional laboratory conditions at the temperature of 20–24°C, relative humidity of 40–70%, and noise level of 60 dB. Each cage was identified using the following data: number of study, group, dose, number, and sex of each animal. Fluorescent lighting provided illumination 12 h per day. Standard good laboratory practice diet and fresh water were provided *ad libitum*. Animal care was in compliance with standard operative procedures of Pharmacology Animal facility, the European convention for the protection of vertebrate animals used for experimental and other scientific purposes (ETS 123). Ethical principles of the study ensured compliance with European Directive 010/63/E, the Law on Amendments to the Animal Protection Act (Official Gazette 37/13, the Animal Protection Act (official Gazette 135/06), Ordinance on the protection of animals used for scientific purposes (Official Gazette 55/13), FELASA recommendations, and recommendations of the Ethics Committee, School of Medicine, University of Zagreb. All experiments received specific approval from the local Ethics Committee at the School of Medicine (the University of Zagreb, Zagreb, Croatia). We randomly assigned 10 rats per experimental group and period for all experiments.

### Drugs

Pentadecapeptide BPC 157 (manufactured by Diagen, Ljubljana, Slovenia (Ilic et al., 2009; Ilic et al., 2010; Ilic et al., 2011a; Ilic et al., 2011b; Gjurasin et al., 2010; Tudor et al., 2010; Klicek et al., 2013; Sikiric et al., 2013; Sikiric et al., 2016; Lojo et al., 2016; Drmic et al., 2017; Medvidovic-Grubisic et al., 2017), GEPPPGKPADDAGLV, M.W. 1,419, partial sequence of human gastric juice protein BPC, peptide with 99% high-pressure liquid chromatography (HPLC) purity, expressing 1-des-Gly peptide as an impurity, freely soluble in water at pH 7.0 and in saline) was dissolved in distilled water. L-NAME (Sigma United States) was commercially purchased. Diazepam (5 mg/kg b.w. i.p.) and sodium thiopental (5 mg/kg b.w. i.p.) were used for anesthesia.

### Procedure

Before retrobulbar application and/or fundus imaging (filmed with USB microscope camera “Veho Discovery VMS-004 Deluxe” and “VOLK” Digital Wide Field Lens for indirect ophthalmoscopy), the animals were deeply anesthetized with diazepam (5 mg/kg b.w. i.p.) and sodium thiopental (5 mg/kg b.w. i.p.), pupils were dilated with tropicamide (Mydriacyl 1% Alcon, United Kingdom) two drops in both eyes, and then anesthetized with tetracaine drops (Tetracaine, Pliva, Zagreb, Croatia) in both eyes.

The retinal damage procedure included L-NAME administration in a total dose of 5 mg/kg, which was given by retrobulbar application in each eye, with 0.5 mg/0.1 ml saline per eye.

Medication regimen, given after L-NAME administration, included a total dose of 10 μg/kg or 10 ng/kg of BPC 157 *via* retrobulbar application in each eye, 1 μg/0.1 ml of saline/each eye or 1 ng/0.1 ml saline/each eye, or saline only *via* retrobulbar application in each eye (0.1 ml/each eye). The application time was either at 20 min after L-NAME administration (as early therapy) or at 48 h after L-NAME administration (as delayed therapy). L-NAME and BPC 157 regimens and dose application were adjusted according to our previous studies (reviewed Sikiric et al., 2014; Kokot et al., 2016).

Alternatively, to perceive the effect of the given medication itself (without L-NAME), rats received only BPC 157 (total dose 10 μg/kg or 10 ng/kg as retrobulbar application in each eye, 1 μg/0.1 ml saline/each eye, or 1 ng/0.1 ml saline/each eye) or only saline *via* retrobulbar application in each eye (0.1 ml/each eye) (as an additional control sham group) at the corresponding time point.

Assessment procedure (the fundus images were filmed for 1 min) included several specific time points as follows: i) normal rats before application; ii) before L-NAME retrobulbar administration and immediately thereafter; iii) when the rats underwent early treatment at 20 min after L-NAME administration: before medication (*a*) and after medication (*b*, *c*, *d*, *e*, *f*, and *g*), immediately after (*b*), at the days 1 (*c*), 2 (*d*), 7 (*e*), 14 (*f*), and 28 (*g*) L-NAME time; iv) these mentioned points were also included in the assessment of the rats which only underwent BPC 157 or saline retrobulbar administration; and v) in the rats, which underwent delayed therapy, at 48 h post–L-NAME administration: immediately before medication (*A*) and after application (*B*, *C*, *D*, *E*, *F*, and *G*), immediately after (*B*), and after 1 and 2 days, corresponding to day 3 (C) and day 4 (*D*) L-NAME time, and at the days 7 (*E*), 14 (*F*), and 28 (*G*) L-NAME time.

During recording, the animals received artificial tears (Isopto Tears, Alcon Pharmaceuticals, United Kingdom) in the eyes. Moistening of the cornea allows better visualization of the fundus and clearer images of the retina.

The degree of ischemia of the retina was scored 1–4 by taking images of the animals fundus as follows: 1: orderly eye background, normal presentation of the choroidal blood vessels (normal reddish fundus background color); 2: discrete generalized irregularity in the diameter of the blood vessels with mild atrophy of the optic disc, normal presentation of the choroidal blood vessels (normal reddish fundus background color); 3: moderate generalized irregularity of the diameter of blood vessels with moderate atrophy of the optic disc, barely visible (faint presentation) choroidal blood vessels (brighter fundus background color); and 4: strong generalized irregularity of the diameter of the blood vessels with severe atrophy of the optic disc, barely visible (extremely faint presentation) choroidal blood vessels (bright fundus background color). The images were processed with software purchased with the USB microscope camera “Veho Discovery VMS-004 Deluxe”.

#### Behavior

The behavior of the animals was recorded from the second day, after the last retrobulbar application. To illustrate animal behavior after retrobulbar application of the agents, each of the rats was mounted on an elevated surface (50 cm long, 20 cm width, and 30 cm high) and filmed for 1 min. We recorded the number of rats that maintained the initial position of firmly standing on the surface with posterior legs, with only limited movements around, as well as the number of those demonstrating no freezing behavior, quick exploration, and escaping from the elevated surface.

### Intraocular Pressure Measuring

Intraocular pressure was measured using a calibrated applanation tonometer Tonopen XL by Reichert Technologies (the transducer was placed on the same spot of the cornea for each measurement). The rats were deeply anesthetized with diazepam (5 mg/kg b.w. i.p.) and sodium thiopental (5 mg/kg b.w. i.p.), and furthermore, tetracaine drops were administered in both eyes. Intraocular pressure was assessed in normal rats, and values between 13 and 19 mmHg were considered normal, in accordance with previous rat data (Mermoud et al., 1994).

To assess the effect of retrobulbar administration itself, the values were assessed before retrobulbar application, after retrobulbar application of saline (0.1 ml/each eye), BPC 157 1 μg/0.1 ml saline/each eye, or L-NAME 0.5 mg/0.1 ml saline/each eye, at 1, 2, 4, 6, 8, and 10 min thereafter.

To evaluate the effect of the L-NAME–induced retinal injury protocol and the medication given after L-NAME administration, intraocular pressure was assessed before L-NAME retrobulbar administration as well as thereafter at described intervals.

For the early treatment at 20 min, we assessed intraocular pressure before medication as well as thereafter at described intervals. Further intraocular pressure assessment was performed at days 1 and 2, and after 1, 2, and 4 weeks.

For the delayed therapy, intraocular pressure was assessed as described above, at 48 h post–L-NAME administration, before medication application, and thereafter at described intervals. We performed further intraocular pressure assessment at 20 min after medication, after days 1 and 2, and after 1, 2, and 4 weeks.

Besides the initial short-lasting increase of the intraocular pressure upon retrobulbar application, intraocular pressure was continuously normal, and the data were not specifically shown.

### Microscopy

In the histopathological evaluation at the end of 1, 2, 3, and 4 weeks, the enucleated eyes were fixed in 4% phosphate-buffered formaline, and transverse sections passing through the optic nerve were obtained. The specimens were processed on paraffin wax, and 5-μm-thick paraffin sections were obtained and stained with hematoxylin and eosin or immunohistochemically for FVIII (Dako, Glostrup, Denmark) using Dako Autostainer, according to the manufacturer’s protocols. For the thickness of the retinal tissue, internal plexiform layer, and internal nuclear layer, the measurements (ISSA program (VAMSTEC, Zagreb, Croatia) from the area of maximal tissue damage detected using semi-serial sections, five high-power fields were randomly selected for analysis, were made with an ocular micrometer at 20 magnification within 0.5 mm from the optic disc. Signs of histopathologic changes such as edema, vacuolar degeneration and pyknosis, and polymorphonucleated leukocyte (PMNL) infiltration were noted if present. The histological examination was performed by the pathologist, who was unaware of the treatment.

### Statistical Analysis

We used Statistica 12.1. for Windows to perform statistical analysis. Distribution of data normality was tested by the Kolmogorov–Smirnov test. The data were expressed as arithmetic mean ± standard deviation (SD) and minimum/medium/maximum. The statistical difference among groups was compared using one-way ANOVA followed by the *post hoc* Student–Newman–Keuls’ test or the Kruskal–Wallis test followed by the *post hoc* Mann–Whitney U test (where appropriate). Qualitative data between the control and treatment groups were analyzed by the Fisher test. The differences between the groups were considered statistically significant if *p* < 0.05.

## Results

In general, the retrobulbar L-NAME application procedure induces only short-lasting mild increase of intraocular pressure. Of note, with normal intraocular pressure, the severe eye damage rapidly appears and persists as an inescapable downhill course. If rats receive BPC 157 therapy, a counteraction occurs. We demonstrated BPC 157 therapy effect by fundoscopy, behavior presentation, and histology.

### Intraocular Pressure

**FIGURE 1:**
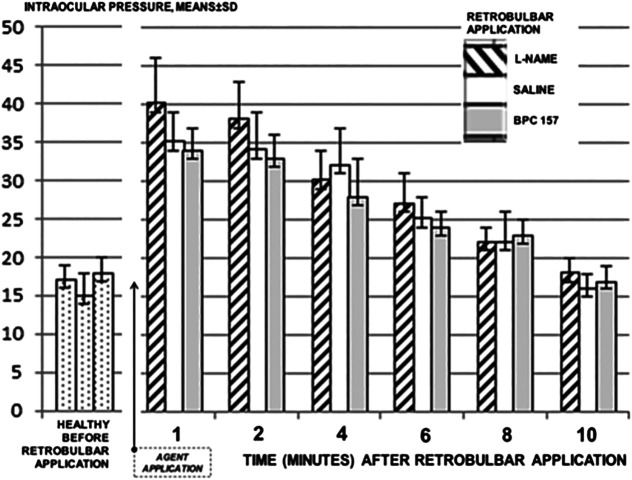
Intraocular pressure was measured using a calibrated applanation tonometer Tonopen XL by Reichert Technologies (the transducer was placed on the same spot of the cornea for each measurement). Intraocular pressure was assessed in normal rats, and values between 13–19 mmHg were considered as normal. Then, the values were assessed before retrobulbar application, and after retrobulbar application of saline (0.1 ml/each eye) (control), BPC 157 1 μg/0.1 ml saline/each eye, or L-NAME 0.5 mg/0.1 ml saline/each eye, at 1, 2, 4, 6, 8, and 10 minutes thereafter. No difference vs. control was noted. 10 rats per experimental group and period for all experiments.

As increased intraocular pressure is an integral feature of ischemic retinal damage in rats, we measured intraocular pressure using a calibrated applanation tonometer. Besides the initial short-lasting increase of intraocular pressure upon retrobulbar application, which appeared equally upon retrobulbar application of saline, L-NAME, and BPC 157 (Figure 1), intraocular pressure was continuously normal, and it was not otherwise changed upon application of BPC 157 or saline (the data are not specifically shown). Thus, all the damages after retrobulbar application of L-NAME appear within the normal range of intraocular pressure in rats.

### Fundoscopy

**FIGURE 2:**
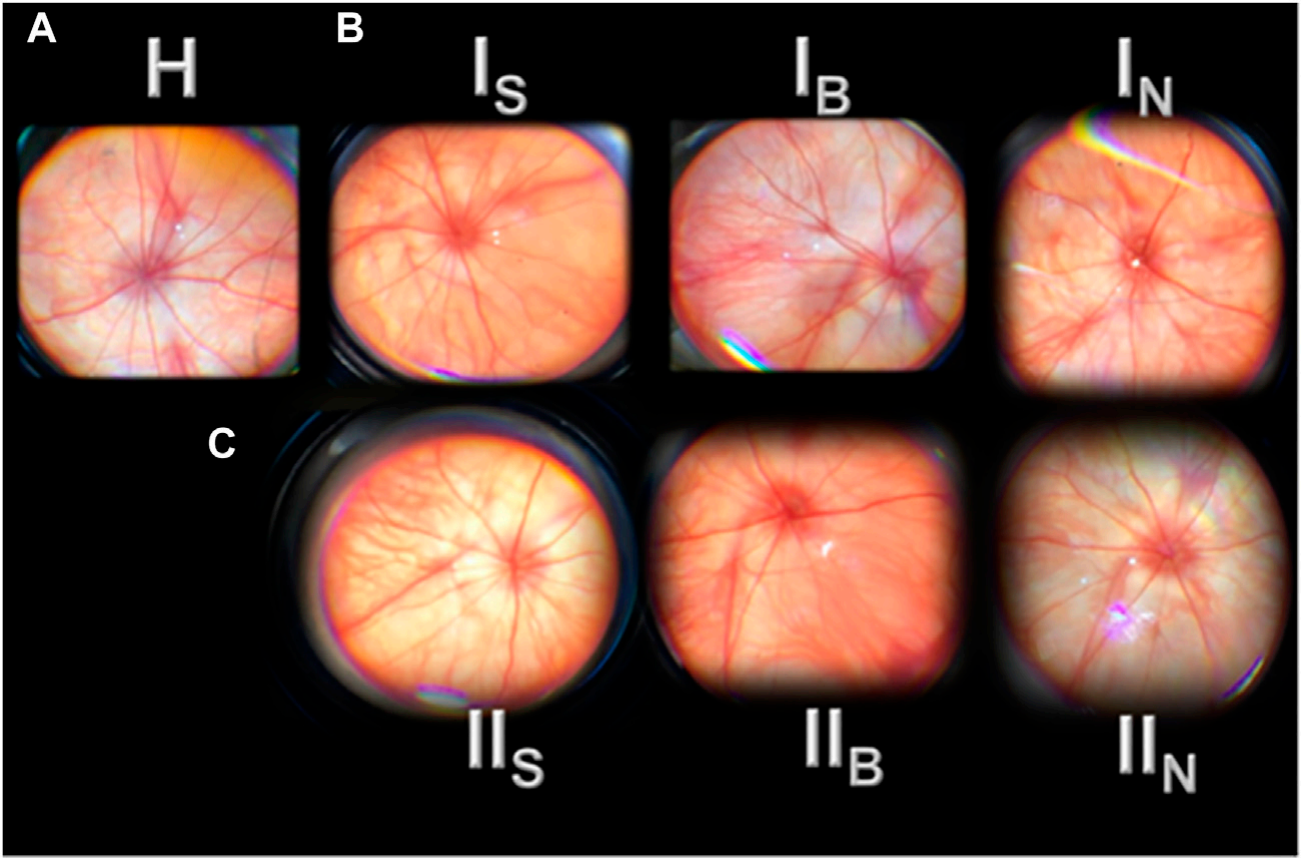
Presentation of the rat retina before (H) and after retrobulbar administered agents (saline (S), BPC 157 (B), and L-NAME (N)), immediately (I), and at 20 min (II) (IS, IB, IN; IIS, IIB, and IIN). H, before agent’s application (H—healthy). Normal eye background, normal presentation of the retinal and choroidal blood vessels **(A)**. I, immediately after agent’s application (IS, IB, and IN) **(B)**. Normal eye background, normal presentation of the retinal and choroidal blood vessels (saline (IS), BPC 157 (IB); moderate generalized irregularity in diameter of blood vessels with moderate atrophy of the optic disk, faint presentation of the choroidal blood vessels (IN). II, 20 min after agent’s application (IIS, IIB, and IIN) **(C)**. Normal eye background, normal presentation of the retinal and choroidal blood vessels (saline (IIS), BPC 157 (IIB) (same presentation maintained till the end of the experiment, data not specifically shown); moderate generalized irregularity diameter blood vessels with moderate atrophy of the optic disk, faint presentation of the choroidal blood vessels (IIN). The images are processed with software purchased with a USB microscope camera “Veho Discovery VMS-004 Deluxe.”

We demonstrated the specific damaging effect of L-NAME application. On the other hand, much like in the healthy rats (Figure 2A), we demonstrated that immediately after saline or BPC 157 administration, there was consistent normal eye background and normal presentation of the retinal and choroidal blood vessels (IS and IB). These remained the same at 20 min (Figure 1) and remained undisturbed until the end of the experiment.

**FIGURE 3:**
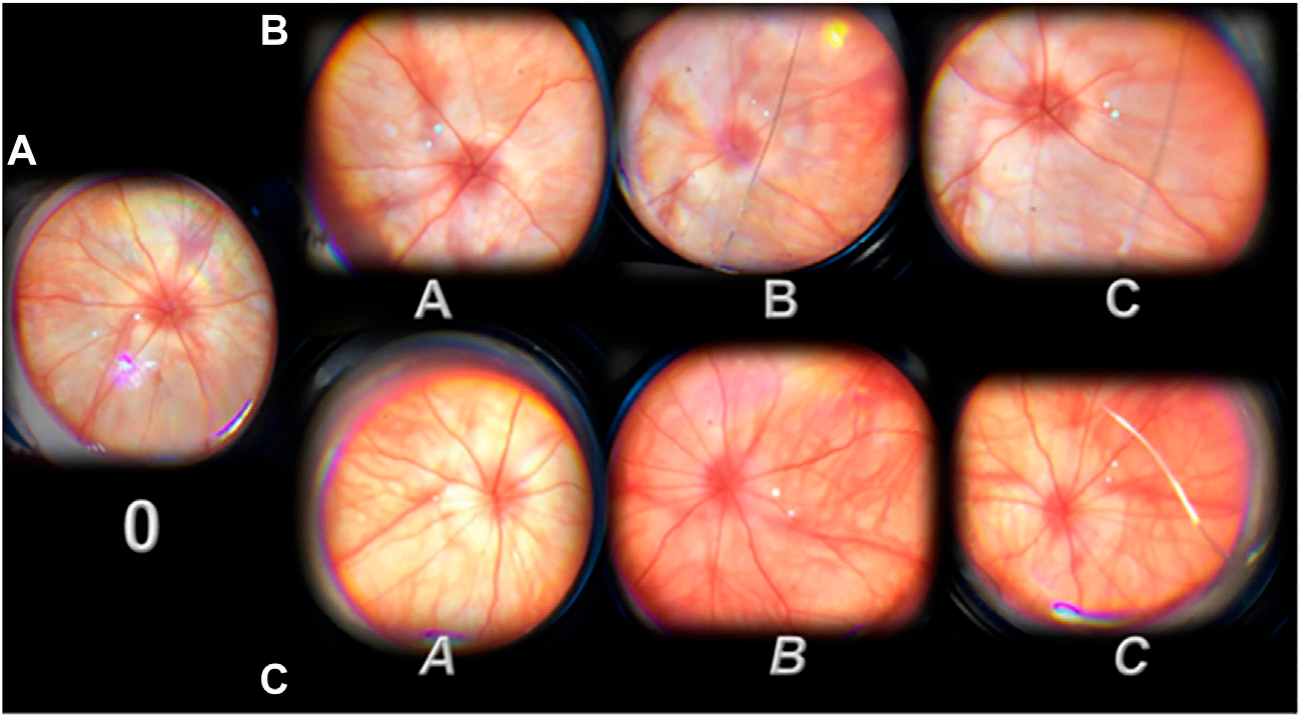
Presentation of the rat retina after retrobulbar administered L-NAME (period from 20 minutes till the 1 day) before (0, **(A)**) and after therapy [saline (A, B, and C; **(B)**); BPC 157 (A, B, and C; **(C)**)] administration. 0, L-NAME point before therapy application, at 20 min after retrobulbar administered L-NAME. Moderate generalized irregularity diameter blood vessels with moderate atrophy of the optic disk, faint presentation of the choroidal blood vessels **(A)**. After therapy application (A, B, and C (saline) or A, B, and C (BPC 157)) **(B,C)**. Immediately after (A, *A*). A. Strong generalized irregularity diameter blood vessels with severe atrophy of the optic disc, extremely poor presentation of the choroidal blood vessels (saline, **B**). *A.* Discrete generalized irregularity diameter blood vessels with mild atrophy of the optic disc, normal presentation of the choroidal blood vessels (BPC 157, **C**). 20 min after (B, *B*). B. Strong generalized irregularity diameter blood vessels with severe atrophy of the optic disc, extremely poor presentation of the choroidal blood vessels (saline, **B**). *B.* Normal eye background, normal presentation of the choroidal blood vessels (BPC 157, **B,C**). 1 day after (C,*C*). C. Strong generalized irregularity diameter blood vessels with severe atrophy of the optic disc, extremely poor presentation of the choroidal blood vessels (saline, **B**). *C*. Normal eye background, normal presentation of the choroidal blood vessels (BPC 157, **C**). The images are processed with software purchased with a USB microscope camera “Veho Discovery VMS-004 Deluxe.”

**FIGURE 4:**
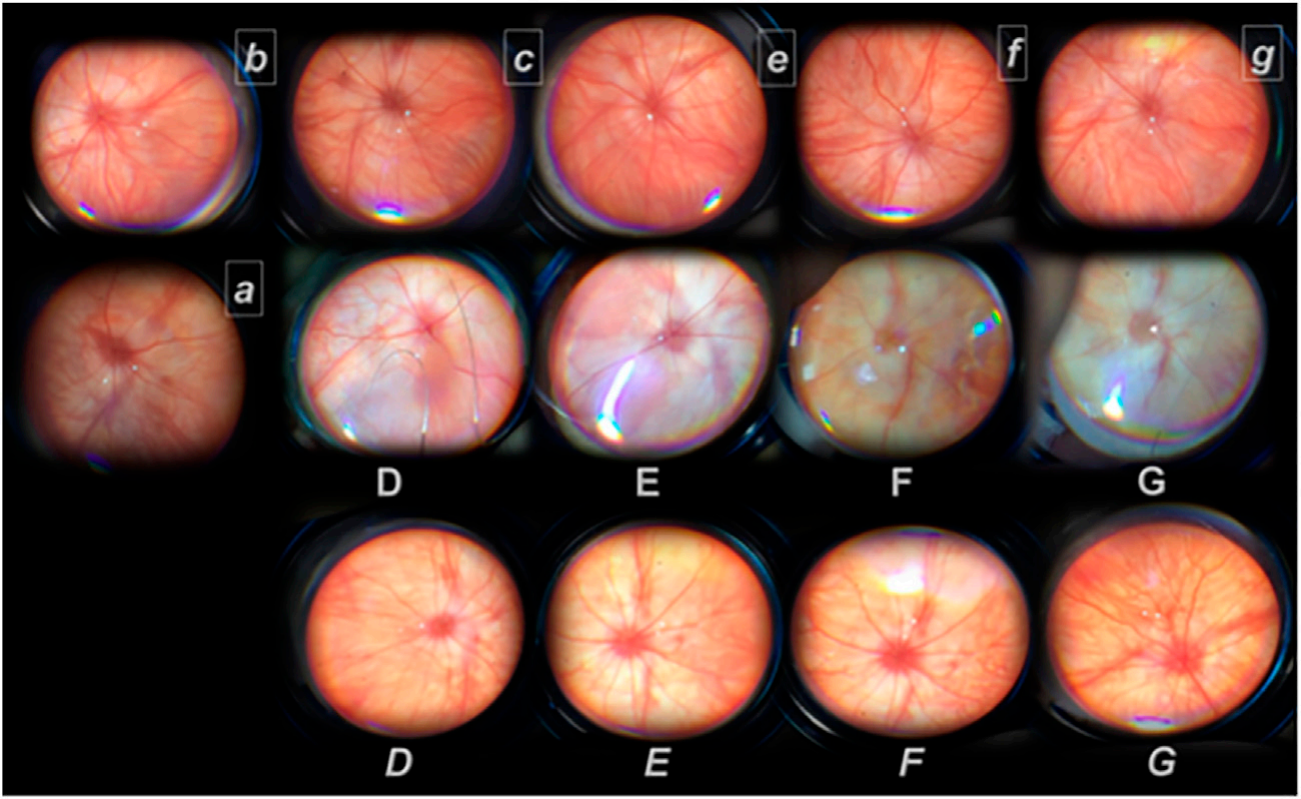
Presentation of the retina after retrobulbar L-NAME application (without therapy (regular font) and with BPC 157 therapy (*italic font*)) from day 2 (D, *D*, *a, and b*) until the end of the experiment (day 3, *c*; week 1 (E, *E*, and *e*); week 2 (F, *F*, and *f*); week 4 (G, *G, and g*)). Rats received. therapy at 20 min (all capitals, D, E, F, and G (saline) or *D, E, F, and G* (*BPC 157*)) or at 48 h after L-NAME (small letters, *a, b, c, d, e, and f* (*BPC 157*)). Presentation of the retina at 2 days after retrobulbar administered L-NAME (D, *D, a, and b*). D. Saline application (at 20 min, or at 48 h after L-NAME). The retina presentation of the rats like in the rats that received L-NAME only (data not specifically shown). Strong generalized irregularity diameter blood vessels with severe atrophy of the optic disc, extremely poor presentation of the choroidal blood vessels. *D.* BPC 157 therapy at 20 min after retrobulbar L-NAME. Normal eye background, normal presentation of the choroidal blood vessels. *a,b,c*. BPC 157 therapy at 48 hours after retrobulbar L-NAME. *a.* Presentation of the retina immediately after retrobulbar BPC 157 application. Discrete generalized irregularity diameter blood vessels with mild atrophy of the optic disc, normal presentation of the choroidal blood vessels. *b.* Presentation of the retina at 20 min after retrobulbar BPC 157 application. Normal eye background, normal presentation of the choroidal blood vessels. *c.* Presentation of the retina at 1 day after retrobulbar BPC 157 application. Normal eye background, normal presentation of the choroidal blood vessels. Presentation of the retina at 1 week after retrobulbar administered L-NAME (E, *E, e*). E. Strong generalized irregularity diameter blood vessels with severe atrophy of the optic disc, extremely poor presentation of the choroidal blood vessels (saline). BPC 157 therapy at 20 minutes (*E*) or at 48 h after retrobulbar L-NAME (e). E, e. Normal eye background, normal presentation of the choroidal blood vessels. Presentation of the retina at 2 weeks after retrobulbar administered L-NAME (F, *F, f*). F. Strong generalized irregularity diameter blood vessels with severe atrophy of the optic disc, extremely poor presentation of the choroidal blood vessels (saline). BPC 157 therapy at 20 minutes (*F*) or at 48 h after retrobulbar L-NAME (*f*).*F, f.* Normal eye background, normal presentation of the choroidal blood vessels. Presentation of retina at 4 weeks after retrobulbar administered L-NAME (G, *G, g*). G. Strong generalized irregularity diameter blood vessels with severe atrophy of the optic disc, extremely poor presentation of the choroidal blood vessels (saline). BPC 157 therapy at 20 min (*G*) or at 48 h after retrobulbar L-NAME (*g*).*G, g*. Normal eye background, normal presentation of the choroidal blood vessels. The images are processed with software purchased with a USB microscope camera “Veho Discovery VMS-004 Deluxe”.

**FIGURE 5:**
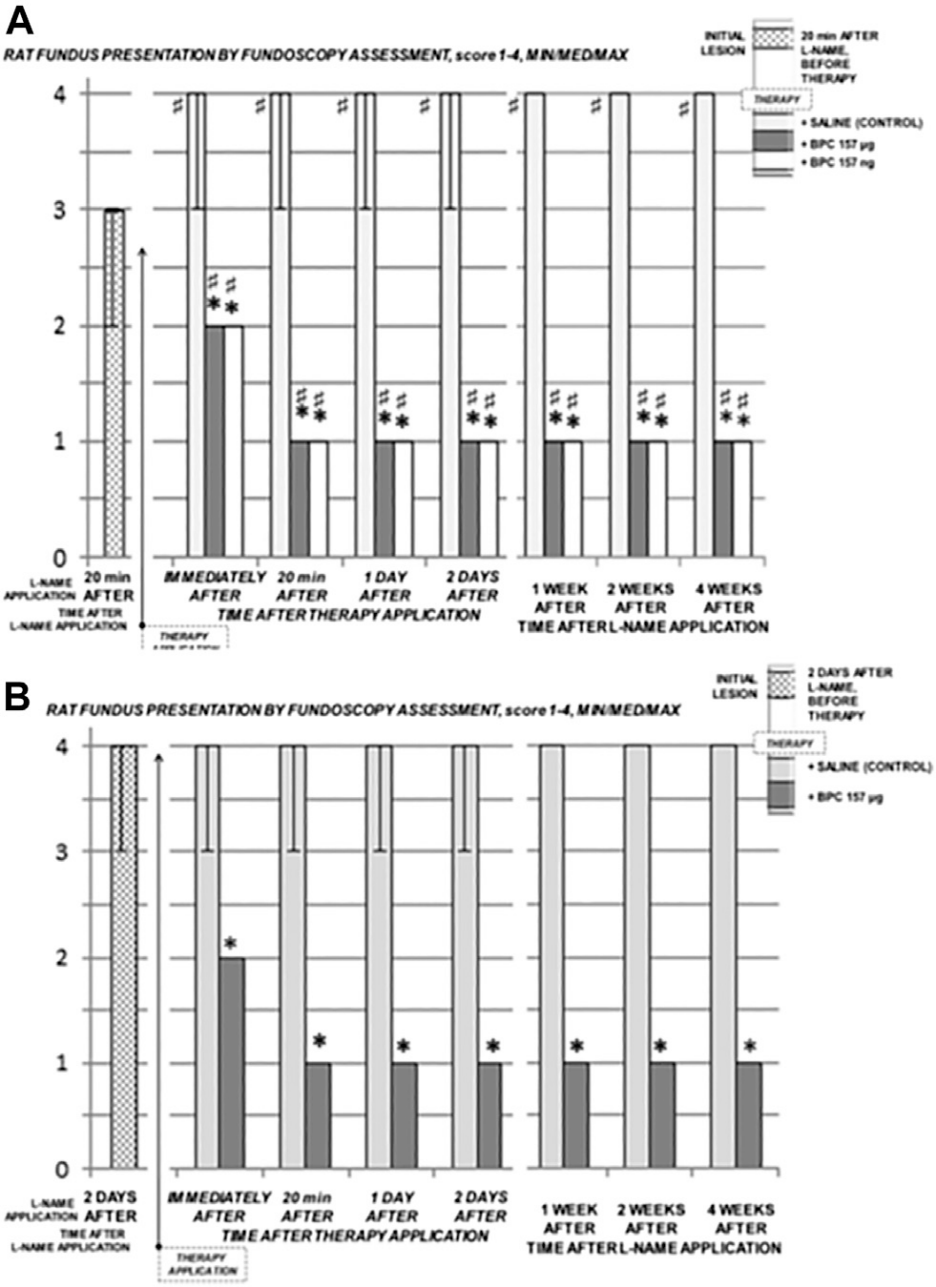
Retrobulbar L-NAME application–induced retinal damage as fundus assessment by fundoscopy. L-NAME retrobulbar application (total dose 5 mg/kg, which was divided by retrobulbar application in each eye, 0.5 mg/0.1 ml saline/eye). Rats underwent L-NAME that received BPC 157 therapy (total dose 10 μg/kg or 10 ng/kg as retrobulbar application in each eye, 1 μg/0.1 ml saline/eye or 1 ng/0.1 ml saline/eye) at 20 min **(A)** or at 2 days **(B)** (BPC 157 μg, BPC 157 ng). Quick demonstration of preserved fundus presentation. Rats underwent L-NAME that received saline as therapy [saline only as a retrobulbar application in each eye (0.1 ml/eye)] (+SALINE). Severely damaged fundus presentation. *p<0.05, at least vs. control; ♯ *p*<0.05, at least vs. initial values. 10 rats per experimental group and period for all experiments.

In contrast, with the administration of L-NAME, there was a moderate generalized irregularity in the diameter of blood vessels with moderate atrophy of the optic disc, faint presentation of the choroidal blood vessels appears immediately, and these lesions (Figure 2, IN, IIN) rapidly progress (Figure 3, A→C; Figure 4; D→G; Figure 5). Subsequently, strong generalized irregularity diameter blood vessels with severe atrophy of the optic disc and extremely poor presentation of the choroidal blood vessels appeared.

BPC 157 retrobulbar application was given as therapy after L-NAME at 20 min or at 48 h, as early therapy (i.e., after L-NAME at 20 min), or as delayed therapy (after L-NAME at 48 h); BPC 157 retrobulbar application stopped this downhill course and instantly reversed back to the normal presentation (Figure 3, A→C; Figure 4 D→G; a→g, Figure 5). The beneficial effect of BPC 157 retrobulbar application seems to be permanent (Figure 3 A→C; Figure 4 D→G; a→g, Figure 5).

### Microscopy

**FIGURE 6:**
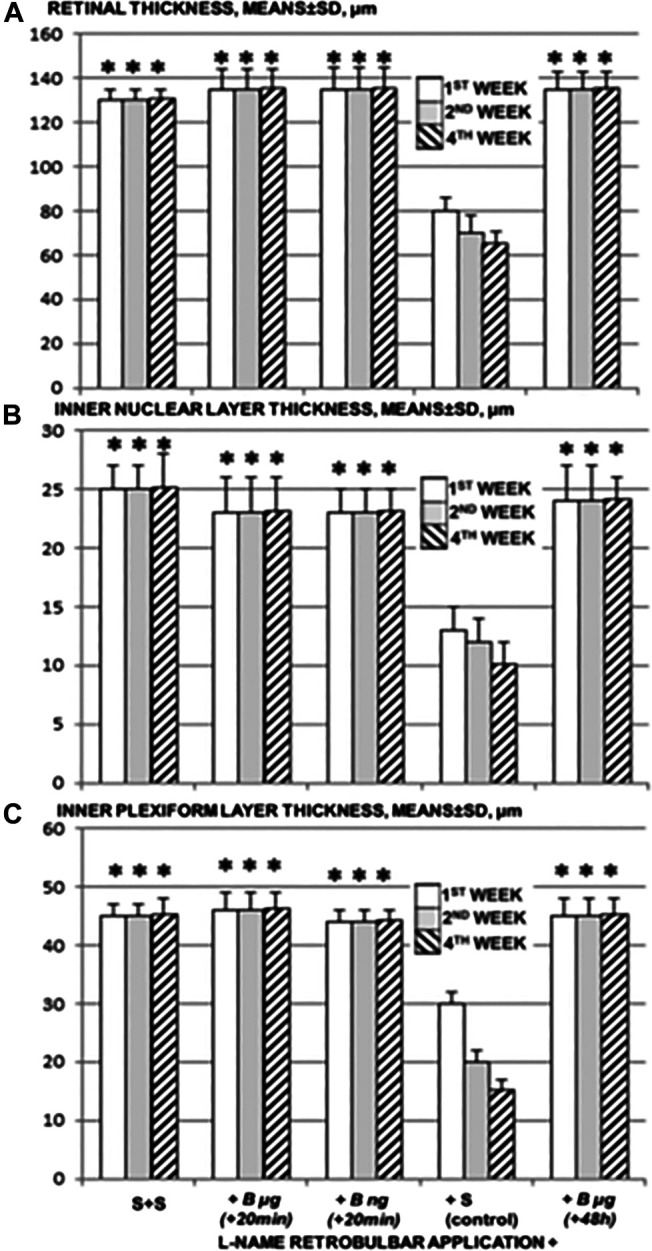
Retrobulbar L-NAME application–induced retinal damage. Retinal thickness **(A)**, inner nuclear layer thickness **(B)**, and inner plexiform layer thickness **(C)** presentation after retrobulbar agent’s application. Left. Saline retrobulbar application(s) completely preserved retinal thickness (S + S). Right. L-NAME retrobulbar application (total dose 5 mg/kg, which was divided by retrobulbar application in each eye, 0.5 mg/0.1 ml saline/eye) (L-NAME retrobulbar application +). Rats underwent L-NAME that received BPC 157 therapy {total dose 10 μg/kg or 10 ng/kg as retrobulbar application in each eye, 1 μg/0.1 ml saline/eye or 1 ng/0.1 ml saline/eye) at 20 min [B μg (+20 min), B ng (+20 min)], or 48 h [B μg (+48 h)]}. Preserved retinal thickness, inner nuclear layer thickness, and inner plexiform layer thickness. Rats underwent L-NAME that received saline as therapy [saline only as a retrobulbar application in each eye (0.1 ml/eye)] [since no different, the rats underwent L-NAME that received saline retrobulbar application and were presented together (+S (control)]. Less retinal thickness. *p<0.05, at least vs. control. 10 rats per experimental group and period for all experiments.

**FIGURE 7:**
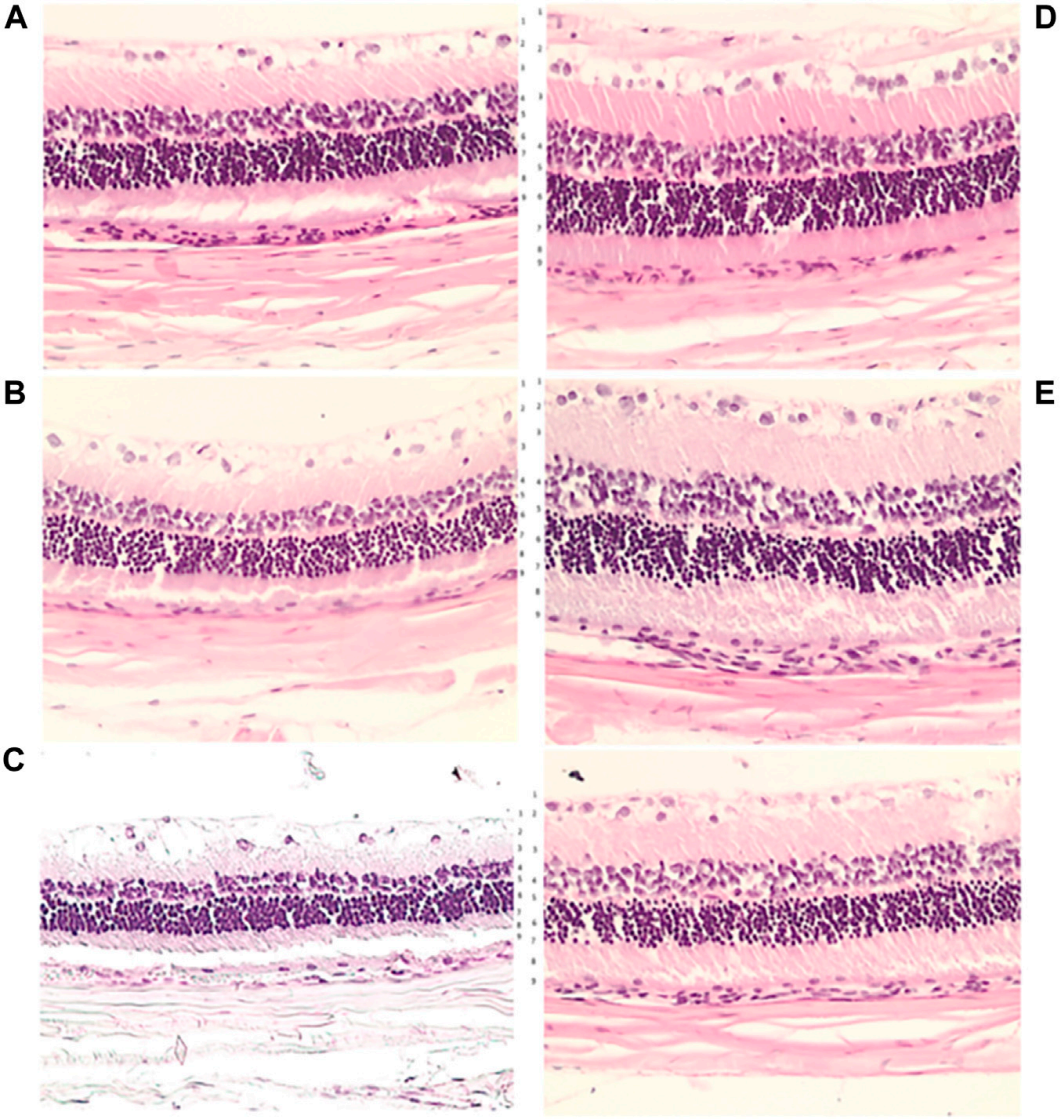
Microscopic presentation of the rat retina at week 1 **(A)**, week 2 **(B)** and week 4 **(C)** after L-NAME retrobulbar application, in controls **(D)** and BPC 157 treated rats **(E)**, HE, x20. Transverse section of the retina (0.4–0.7 mm on the temporal side of the optic disc) showing a strict difference in the retinal layers and full retina thickness in the rats underwent L-NAME that received retrobulbar saline application and those that received BPC 157. More regular inner and outer nuclear layer and more regular distribution of ganglion cells, preserved thickness of the retina, the inner plexiform layer and inner nuclear layer at week 1 **(A,E)**. More regular inner and outer nuclear layer and more regular distribution of ganglion cells, preserved thickness of the retina, the inner plexiform layer and inner nuclear layer at week 2 **(B,E)** (more regular inner and outer nuclear layer and more regular distribution of ganglion cells, preserved thickness of retina, the inner plexiform layer and inner nuclear layer). At week 4, BPC 157 treated rats show the preserved thickness of the whole retina, also inner plexiform layer and inner nuclear layer **(C,E)**. Contrarily, degeneration of ganglion cells in control group is the most evident **(C,D)**. Also, the outer nuclear layer is more regular in BPC 157 treated rats. 1—internal limiting membrane; 2—nerve fiber and ganglion cell layers; 3—inner plexiform layer; 4—inner nuclear layer; 5—outer plexiform layer; 6—outer nuclear layer; 7—outer limiting membrane; 8—photoreceptor layer; 9—pigment epithelium.

Retrobulbar application of L-NAME resulted in degeneration of ganglion cells and in nerve cell layer narrowing of the blood vessel lumen (using immunohistochemistry for Factor VIII). There was particularly damaged inner plexiform and inner nuclear layer, less thickness, along with complete retinal damage, less thickness, noted 1 week after L-NAME retrobulbar administration. These layers progressively became more damaged toward the end of the 2 and 4 weeks (Figure 6, Figure 7).

BPC 157 retrobulbar application was given as therapy after L-NAME at 20 min or at 48 h. Along with the beneficial effect noted with fundoscopy assessment, all BPC 157 rats (both μg- and ng-regimen, those that received medication at 20 min after L-NAME, and those that received medication at 48 h after L-NAME) commonly presented preserved plexiform and inner nuclear layer and retinal thickness (Figure 6, Figure 7). They did not show narrowing of the blood vessel lumen.

### Behavior

**FIGURE 8:**
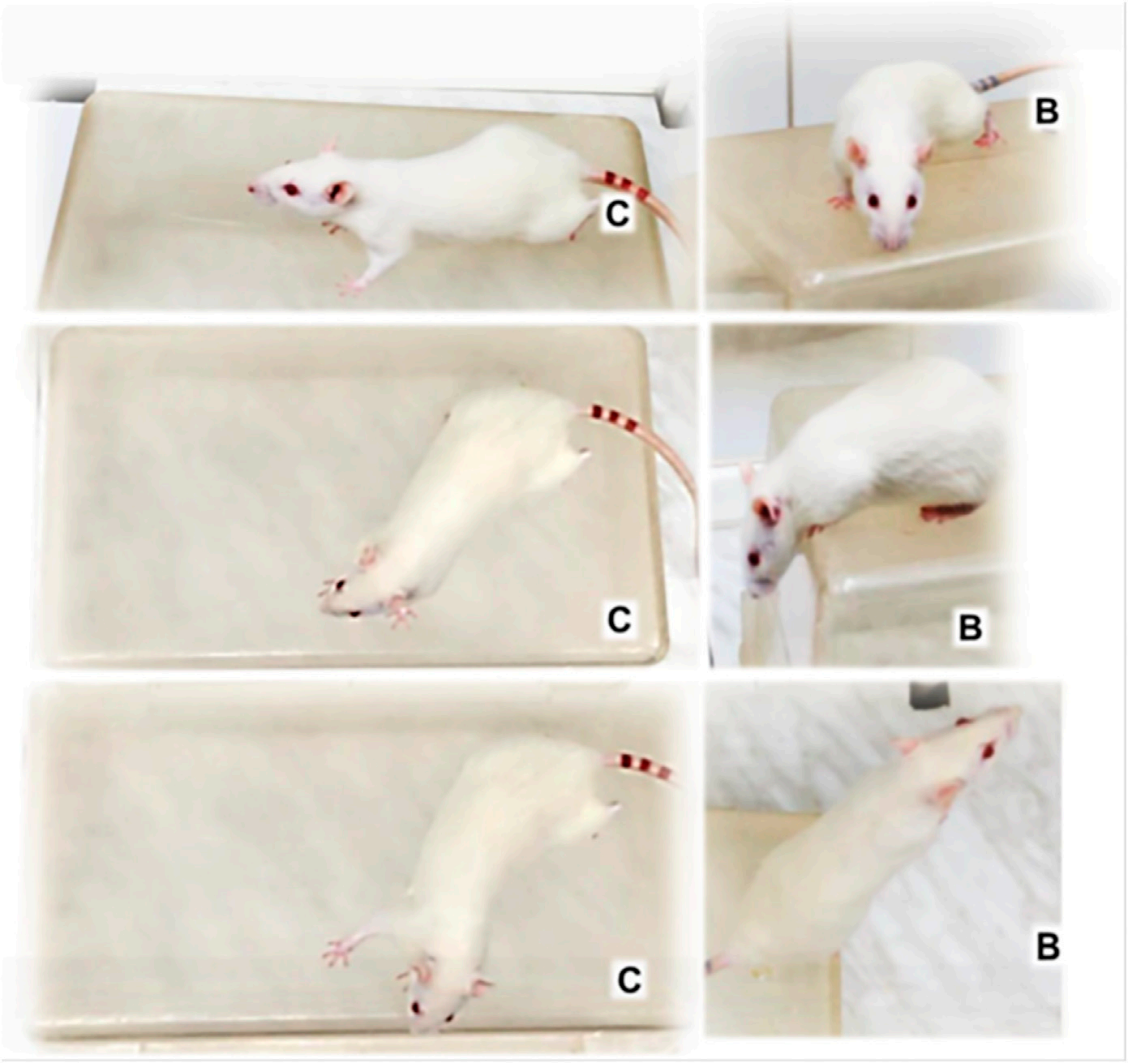
Illustrative animal behavior days after retrobulbar application of L-NAME when mounted on an elevated surface (50 cm long, 20 cm width, 30 cm high) and filmed for 1 minute. Regularly, rats maintained the initial position firmly standing on to the surface with posterior legs, almost―frozen‖, with only limited movements around [control (C)], unless treated with BPC 157 (B) showing no freezing behavior, but quick exploration, and escaping from the elevated surface.

**FIGURE 9:**
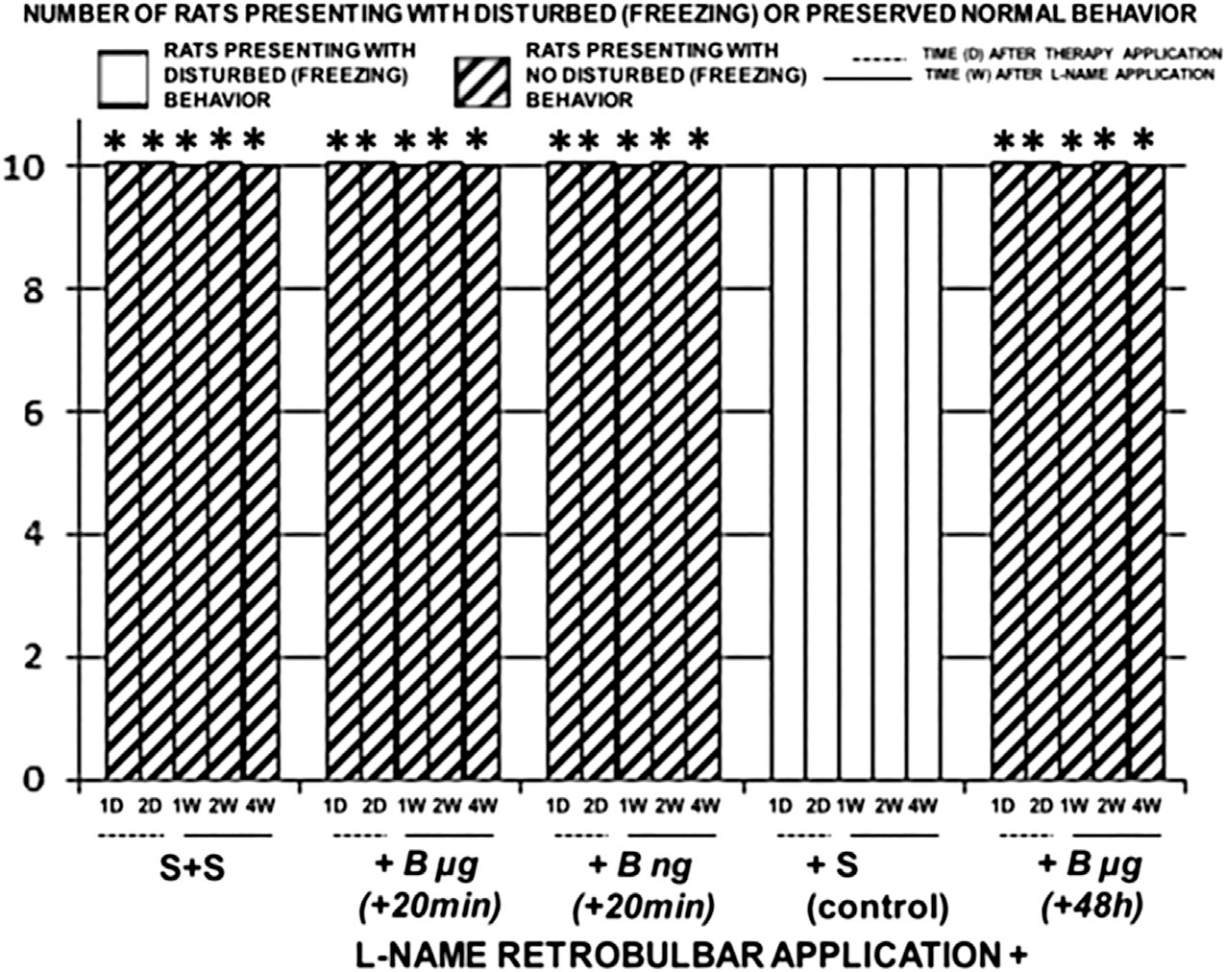
Retrobulbar L-NAME application induced retinal damage. Rats behavior presentation after retrobulbar agent’s application. Left. Saline retrobulbar application(s) completely preserved rats behavior and they did not present freezing behavior while investigated (S + S). Right. L-NAME retrobulbar application (total dose 5 mg/kg, which was divided by the retrobulbar application in each eye, 0.5 mg/0.1 ml saline/eye) (L-NAME retrobulbar application +). Rats underwent L-NAME that received BPC 157 therapy (total dose 10 μg/kg or 10 ng/kg as retrobulbar application in each eye, 1 μg/0.1 ml saline/eye or 1 ng/0.1 ml saline/eye) at 20 min [B μg (+20 min), B ng (+20 min)], or 48 h [B μg (+48 h)]. Rats presented behavior without any freezing behavior. Rats underwent L-NAME that received saline as therapy [saline only as retrobulbar application in each eye (0.1 ml/eye)] [since no different the rats underwent L-NAME that received saline retrobulbar application were presented together (+S (control)]. Rats continuously presented mostly freezing behavior while investigated. *p<0.05, at least vs. control. 10 rats per experimental group and period for all experiments.

Illustrative animal behavior on days and weeks after retrobulbar application of L-NAME when mounted on an elevated surface was that rats maintained the initial position of firmly standing on to the surface with posterior legs, almost “frozen,” with limited movements only (Figure 8, Figure 9).

BPC 157 retrobulbar application was given as therapy after L-NAME at 20 min or at 48 h. By contrast, BPC 157–treated rats demonstrated no freezing behavior, but quick exploration and escaping from the elevated surface (Figure 8, Figure 9).

## Discussion

With the role of the NO-system (Cudeiro and Rivadulla, 1999; Toda and Nakanishitoda, 2007; Guthrie and Kang-Mieler, 2014) and consequent role of the retrobulbar application of the NOS-blocker L-NAME in rats, there is a particular noxious chain of events invariably leading to rapid severe retinal damage and function failure. Likewise, providing the high range of the stable gastric pentadecapeptide BPC 157 regimens (for review, see Sikiric et al., 2014; Sikiric et al., 2020), there is a consistent evidence that BPC 157 application, given after L-NAME challenge, may instantly break this chain of events, both at its early stage and an already advanced stage. Thus, in addition to the general BPC 157 interaction with the NO-system and counteraction of the adverse effects of NO-agents [including prolonged miosis in rats and guinea pigs (for review, see Sikiric et al., 2014; Kokot et al., 2016)], these particular beneficial findings may also indicate a specific counteraction of the L-NAME–induced noxious events, leading to rat retinal ischemia. Applied after L-NAME, BPC 157 administration would equally oppose the L-NAME vascular effect, and consequences thereof, at the early stage, that is, 20 min after L-NAME application (and also, still the presence of L-NAME at that time (Avontuur et al., 1998)), as well as at 48 h, at the already advanced course, with already more established lesions. It means that BPC 157, in accordance with its particular effect on the NO-system (Hsieh et al., 1997; Sikiric et al., 2014), may reestablish NO-system functioning in the retinal injury, which may be an early injury or lately, a protracted injury, both counteracted.

Unless therapy was given, we demonstrated the characteristic chain of noxious events after L-NAME in all rats. L-NAME rats exhibited the moderate generalized irregularity in the diameter of blood vessels with moderate atrophy of the optic disc, faint presentation of the choroidal blood vessels appearing immediately, and these lesions (Figure 2, IN, IIN), and a rapid progress to the severe stage (Figure 3, A→C; Figure 4 D→G; Figure 5). This appeared as a direct consequence of the retrobulbar L-NAME application. Thereby, particularly damaged inner plexiform and inner nuclear layer, noted at week 1, and thereby thinned retina, appear as a result of the L-NAME course. Together, providing the innate harmful effect of NOS blocker L-NAME itself, these findings may emphasize the positive significance of NO in protecting retinal neurons (and thereby, harmful effect of NOS-blockers (Ostwald et al., 1995, 1997; Imai et al., 1997; Hangai et al., 1999; Sakamoto et al., 2006)). This may override the NO-negative significance (overproduction of NO interacting with oxygen radicals leads to the death of retinal neurons, and thereby, beneficial effect of NOS blocker (Adachi et al., 1998; Ju et al., 2000)).

Furthermore, it was demonstrated that NO is continuously produced in the retina and essential for maintenance of retinal blood flow (Nagaoka et al., 2002). Likewise, it was shown that without affecting main blood pressure, intravitreous L-NAME application in cats had a detrimental retinal effect that started at 40 min (i.e., decreased retinal blood flow and decrease of vessel diameters at 100 min), lasting for 180 min (Nagaoka et al., 2002). Thus, it seems that with retrobulbar application of L-NAME in rats and mentioned disturbances, this harmful effect is even stronger and appears quite immediately. L-NAME retrobulbar application produced a definitive particular vascular defect in the retina responsible for the further damaging course. This course goes, as ophthalmoscopy showed, with quickly disabled arteries, with the central retinal artery affected first, and then the central retinal vein along with the central retinal artery (Hayreh et al., 1978; Osborne et al., 2004). The subsequent poor presentation of the choroidal blood vessels (and thereby ciliary arteries affected as well) would appear as a further progress of damaging course of events. Of note, the evidence that the damaged inner plexiform layer and inner nuclear layer are supplied through the central retinal artery verifies that L-NAME (i.e., vasoconstriction) directly affected the central retinal artery for a firm time to produce/initiate the chain of damaging events. Further progress (i.e., rat retinal thickness, inner plexiform, and inner nuclear layer thickness from week 1 to week 2 to week 4) may indicate the progressive NO-system failure. Evidently, this initial L-NAME–induced defect and subsequent course prompted a particular chain of events. As mentioned above, this may be comparable to the complete occlusion (ligation)-induced defects produced in the other vessels and consequent syndromes (Vukojevic et al., 2018; Gojkovic et al., 2020; Kolovrat et al., 2020). In particular experiments, the infrarenal inferior caval vein (Vukojevic et al., 2018), suprahepatic inferior caval vein (Gojkovic et al., 2020), superior anterior pancreaticoduodenal vein (Amic et al., 2018), portal vein and hepatic artery (Kolovrat et al., 2020), and left colic artery and vein (Duzel et al., 2017) were occluded. Consequently, we noted portal and caval hypertension, aortal hypotension, ECG disturbances, arterial and venous thrombosis, and organ lesions (Vukojevic et al., 2018; Gojkovic et al., 2020; Kolovrat et al., 2020). Therefore, confronted with vessel occlusion (Duzel et al., 2017; Amic et al., 2018; Drmic et al., 2018; Vukojevic et al., 2018; Cesar et al., 2020; Gojkovic et al., 2020; Kolovrat et al., 2020), BPC 157 therapy effect was ascribed to the rapid opening of the collateral pathways to reestablish blood flow and compensate permanent occlusion of the vessel. Accordingly, the concomitant syndromes were fully counteracted (Vukojevic et al., 2018; Gojkovic et al., 2020; Kolovrat et al., 2020). The BPC 157 therapy effect may be analogous in the L-NAME-rats, confronted with the particular damaging retinal effect of L-NAME, and thereby with the particular local effect.

Thereby, while the specific mechanism remains to be further elucidated, a rapid recovering effect on the events going on in the central retinal artery in L-NAME retrobulbar rats may also be possible. Further argument that BPC 157 had conducted a prompt counteraction in the face of L-NAME retinal ischemia may be the mentioned, quite general, counteracting potential of BPC 157 on the L-NAME–induced damaging effects in different models and species (Sikiric et al., 2014). Evidently, BPC 157 counteracted those associated with the arrhythmias and hypertension (Sikiric et al., 1997; Balenovic et al., 2009; Barisic et al., 2013), vascular injuries (Drmic et al., 2017, 2018; Duzel et al., 2017; Amic et al., 2018), pulmonary hypertension (Grabarevic et al., 1997), and pro-thrombotic effect (Stupnisek et al., 2015). Also, BPC 157 counteracted L-NAME–induced prolonged miosis (Kokot et al., 2016) and sphincter dysfunction (Djakovic et al., 2016; Belosic Halle et al., 2017; Becejac et al., 2018), muscle weakness (Medvidovic-Grubisic et al., 2017), and catalepsy (Zemba Cilic et al., 2021). Finally, BPC 157 counteracted L-NAME induced many organ lesion aggravation, mostly associated with free radical formation (Lojo et al., 2016; Luetic et al., 2017; Sucic et al., 2019). Furthermore, from the BPC 157–L-NAME retinal ischemia evidence’s point of view, the ideal solution to the combination of these effects was precisely the chosen L-NAME and BPC 157 protocol in agreement with previous studies in all respects similar to the noted outcome. This common protocol point (and thereby, regular counteraction of the L-NAME effects by BPC 157 administration) may effectively integrate the BPC 157–L-NAME–NO-system relationship in the present retinal ischemia study (Sikiric et al., 1997; Balenovic et al., 2009; Barisic et al., 2013; Stupnisek et al., 2015; Djakovic et al., 2016; Kokot et al., 2016; Lojo et al., 2016; Belosic Halle et al., 2017; Drmic et al., 2017, 2018; Duzel et al., 2017; Luetic et al., 2017; Medvidovic-Grubisic et al., 2017; Amic et al., 2018; Becejac et al., 2018; Sucic et al., 2019; Zemba Cilic et al., 2021). Likewise, BPC 157 induced the NO-release of its own, which is resistant to L-NAME (Sikiric et al., 1997; Turkovic et al., 2004). Also, BPC 157 (in aortal ring) induced vasodilatation (even when endothelium is removed) and regulated vasomotor tone through BPC 157–specific activation of the Src–Caveolin-1–ensdothelial NOS (eNOS) pathway (Hsieh et al., 1997). Activation of the VEGFR2-Akt-eNOS signaling pathway goes without the need of other known ligands or shear stress (Hsieh et al., 2017). In addition, BPC 157 may act as a free radical scavenger (Belosic Halle et al., 2017; Duzel et al., 2017; Luetic et al., 2017; Amic et al., 2018; Drmic et al., 2018; Vukojevic et al., 2018; Sever et al., 2019; Sucic et al., 2019; Kolovrat et al., 2020; Park et al., 2020). In particular, BPC 157 reestablished NO-values in the occluded inferior caval vein and ischemic colon tissue, as well as counteracted increased MDA values (Duzel et al., 2017; Vukojevic et al., 2018). Accordingly, there is an important common understanding that any form of treatment that can enhance the natural cellular antioxidant defense system should have a neuroprotective action in retinal ischemia (Osborne et al., 2004). Finally, the role of BPC 157 as the stabilizer of cellular junction is well founded (Park et al., 2020). It acts *via* increasing tight junction protein ZO-1 expression and transepithelial resistance. Specifically, BPC 157 inhibited the mRNA of inflammatory mediators (iNOS, IL-6, IFNγ, and TNF-α), increased the expression of HSP 70 and 90, and antioxidant proteins (such as HO-1, NQO-1, glutathione reductase, glutathione peroxidase 2, and GST-pi) (Park et al., 2020). Consequently, it may effectively counteract ischemia-induced increased capillary permeability also in the rats damaged by retrobulbar L-NAME administration.

Further, we should appreciate the noted methodology point (i.e., the vascular failure obtained by retrobulbar L-NAME injection and by therapy counteracted, as specific major findings, and the normal intraocular pressure—the severe eye damage). This would exclude the possible nonspecific contribution of the additional volume (as it may be the infusion into the anterior chamber to increase intraocular pressure above systolic pressure to induce ischemia (Ostwald et al., 1995,^1997^; Imai et al., 1997; Sakamoto et al., 2006)). Upon drug retrobulbar application, intraocular pressure increase was very transitory in all rat groups. The changes occurred only in L-NAME rats, while normal eye background and no change in the normal presentation of the retinal and choroidal blood vessels were observed in the rats that received saline (IS) or BPC 157 (IB) as sham treatment. With the advanced L-NAME time, that is, 20 min or 48 h, the specific vascular failure points the prompt aggravation immediately after retrobulbar saline application and prompt recovering effect after retrobulbar BPC 157 application. This would also exclude the possible contribution of the applied concomitant procedure and given agents on the intraocular pressure. Otherwise, different effects were reported for the agents used in anesthesia {diazepam [increase (Kovalcuka et al., 2013)] and sodium thiopental [decrease (Alipour et al., 2014)]}, for mydriasis (tropicamide, a short-acting anticholinergic agent [increase (Portney and Purcell, 1975)], and local anesthesia {tetracaine drops [increase (Faghihi et al., 2020) or decrease (Sarchahi and Bozorgi, 2012)]}.

Finally, the obtained findings should be summarized with the previous eye evidence. It may be found that BPC 157 participates in ocular control (i.e., BPC 157 counteracted L-NAME-miosis and atropine-mydriasis) (Kokot et al., 2016). This effect goes *via* NO-mediated and cholinergic mechanisms (Kokot et al., 2016), which were accepted as key in ophthalmic arterial dilatation (Osborne et al., 2004). This may be the common point for the transparency maintained, fully rescued total debridement of corneal epithelium (Lazic et al., 2005), and perforating corneal incisions (Masnec et al., 2015), and thereby antagonization of L-NAME–induced retinal ischemia. Thereby, as L-NAME uniformly affected all control rats, along with the fundoscopic and microscopic findings, the final clue may be the highly expressed freezing behavior in all control rats, which was completely recovered after BPC 157 therapy was given. In support, we evidenced the pentadecapeptide BPC 157 (Sikiric et al., 2013; Sikiric et al., 2016) centrally mediated counteraction of the L-NAME–induced catalepsy as well as BPC 157, L-NAME, L-arginine, and NO-relation, in the suited acute and chronic rat behavioral models resembling “positive-like” symptoms of schizophrenia (Zemba Cilic et al., 2021). In terms of the suited rat behavioral models, we mentioned the counteracting potential of BPC 157 on both dopamine- (Zemba Cilic et al., 2021) and NO-system–induced central disturbances (Zemba Cilic et al., 2021). This can explain the reversed freezing rat behavior as consequent to the L-NAME–induced retinal ischemia, which was recovered by BPC 157 administration. In principle, this may be analogous to the demonstrated BPC 157–NO-system relations in many models and species (see Balenovic et al., 2009; Barisic et al., 2013; Sikiric et al., 2014; Kokot et al., 2016; Lojo et al., 2016; Belosic Halle et al., 2017; Drmic et al., 2017, 2018; Duzel et al., 2017; Luetic et al., 2017; Medvidovic-Grubisic et al., 2017; Amic et al., 2018; Sucic et al., 2019; Zemba Cilic et al., 2021). Besides, BPC 157 therapy effect always exhibited the functional recovery as well. This was noted with peripheral nerve injury (Gjurasin et al., 2010) and central nervous disturbances (i.e., traumatic brain injury (Tudor et al., 2010), various encephalopathies (Ilic et al., 2009; Ilic et al., 2010; Ilic et al., 2011a; Ilic et al., 2011b; Klicek et al., 2013; Lojo et al., 2016; Drmic et al., 2017; Medvidovic-Grubisic et al., 2017), stroke (Vukojevic et al., 2020), and spinal cord injury (Perovic et al., 2019)). Likewise, there are many functional and structural similarities between the retina and brain (Osborne et al., 2004), and BPC 157 can likely cross the blood–brain barrier to induce neurotransmitter release (Tohyama et al., 2004; Sikiric et al., 2016).

On the other hand, for the L-NAME retrobulbar application–induced retinal ischemia model and BPC 157 therapy in rats, we should emphasize the point that animal studies *per se* may be cautious regarding their results, although the role of an animal model is indispensable (Minhas et al., 2015; Vestergaard et al., 2019). Also, we should emphasize the relative paucity of the BPC 157 clinical data (Seiwerth et al., 2018; Sikiric et al., 2018; Sikiric et al., 2020; Gwyer et al., 2019). However, it should be noted that BPC 157 was proven to be efficacious in the ulcerative colitis, both in clinical settings (Veljaca et al., 2003; Ruenzi et al., 2005) and in experimental rat studies (see Duzel et al., 2017), and has very safe profile (LD1 could be not achieved) (Seiwerth et al., 2018), a point recently confirmed (Xu et al., 2020). Thus, while the additional studies will be done, we could suggest, based on the role of NO-system (Cudeiro and Rivadulla, 1999; Toda and Nakanishitoda, 2007; Guthrie and Kang-Mieler, 2014), known BPC 157–NO-system close relations (Sikiric et al., 2014; Hsieh et al., 2017), and consequent role of the retrobulbar application of the NOS-blocker L-NAME and BPC 157 therapy in rats, that BPC 157 may consistently counteract retinal ischemia in rats. Hopefully, providing that it may mimic the occlusion of the central retinal artery in patients (Minhas et al., 2015; Chronopoulos and Schutz, 2019), all together, we can envisage the further clinically effective treatments of the retinal ischemia using the stable gastric pentadecapeptide BPC 157.

## Data Availability

The raw data supporting the conclusion of this article will be made available by the authors, without undue reservation.

## References

[B1] Adachi K. Kashii S. Masai H. Ueda M. Morizane C. Kaneda K. (1998). Mechanism of the Pathogenesis of Glutamate Neurotoxicity in Retinal Ischemia. Graefesarch Clin. Exp. Ophthalmol. 236 (10), 766–774. 10.1007/s004170050156 9801892

[B2] Alipour M. Derakhshan A. Pourmazar R. Abrishami M. Ghanbarabadi V. G. (2014).Effects of Propofol, Etomidate, and Thiopental on Intraocular Pressure and Hemodynamicresponses in Phacoemulsification by Insertion of Laryngeal Mask Airway. J. Ocul. Pharmacolther. 30 (8), 665–669. 10.1089/jop.2013.0165 24991995

[B3] Amic F. Drmic D. Bilic Z. Krezic I. Zizek H. Peklic M. (2018). Bypassingmajor Venous Occlusion and Duodenal Lesions in Rats, and Therapy with the Stable Gastricpentadecapeptide BPC 157, L-NAME and L-Arginine. World J. Gastroenterol. 24 (47), 5366–5378. 10.3748/wjg.v24.i47.5366 30598581 PMC6305534

[B4] Avontuur J. A. M. Buijk S. L. C. E. Bruining H. A. (1998). Distribution and Metabolism of N G -nitro- L -arginine Methyl Ester in Patients with Septic Shock. Eur. J. Clin. Pharmacol. 54 (8), 627–631. 10.1007/s002280050525 9860150

[B5] Balenovic D. Bencic M. L. Udovicic M. Simonji K. Hanzevacki J. S. Barisic I. (2009). Inhibition of Methyldigoxin-Induced Arrhythmias by Pentadecapeptide BPC 157:A Relation with NO-System. Regul. Pept. 156 (1-3), 83–89. 10.1016/j.regpep.2009.05.008 19465062

[B6] Barisic I. Balenovic D. Klicek R. Radic B. Nikitovic B. Drmic D. (2013). Mortal Hyperkalemia Disturbances in Rats Are NO-System Related. The Life Saving Effect Ofpentadecapeptide BPC 157. Regul. Pept. 181, 50–66. 10.1016/j.regpep.2012.12.007 23327997

[B7] Becejac T. Cesarec V. Drmic D. Hirsl D. Madzarac G. Djakovic Z. (2018). An Endogeous Defensive Concept, Renewed Cytoprotection/adaptive Cytoprotection: Intra(per)-Oral/intragastric strong Alcohol in Rat. Involvement of Pentadecapeptide BPC 157 and Nitric Oxide System. J. Physiol. Pharmacol. 69 (3). 10.26402/jpp.2018.3.11. 10.26402/jpp.2018.3.11 30279308

[B8] Belosic Halle Z. Vlainic J. Drmic D. Strinic D. Luetic K. Sucic M. (2017). Class Side Effects: Decreased Pressure in the Lower Oesophageal and the Pyloric Sphinctersafter the Administration of Dopamine Antagonists, Neuroleptics, Anti-emetics, L-NAME, Pentadecapeptide BPC 157 and L-Arginine. Inflammopharmacology 25, 511–522. 10.1007/s10787-017-0358-8 28516373

[B9] Cesar L. B. Gojkovic S. Krezic I. Malekinusic D. Zizek H. Vuletic L. B. (2020). Bowel Adhesion and Therapy with the Stable Gastric Pentadecapeptide BPC 157, L-NAME and L-Arginine in Rats. World J. Gastrointest. Pharmacol. Ther. 11 (5), 93–109. 10.4292/wjgpt.v11.i5.93 33251034 PMC7667405

[B10] Chang C. H. Tsai W. C. Hsu Y. H. Pang J. H. S. (2014). Pentadecapeptide BPC 157enhances the Growth Hormone Receptor Expression in Tendon Fibroblasts. Molecules 19 (11), 19066–19077. 10.3390/molecules191119066 25415472 PMC6271067

[B11] Chang C. H. Tsai W. C. Lin M. S. Hsu Y. H. Pang J. H. S. (2011). The Promotingeffect of Pentadecapeptide BPC 157 on Tendon Healing Involves Tendon Outgrowth, Cellsurvival, and Cell Migration. J. Appl. Physiol. 110 (3), 774–780. 10.1152/japplphysiol.00945.2010 21030672

[B12] Chronopoulos A. Schutz J. S. (2019). Central Retinal Artery Occlusion-A New, Provisional Treatment Approach. Surv. Ophthalmol. 64 (4), 443–451. 10.1016/j.survophthal.2019.01.011 30707925

[B13] Cudeiro J. Rivadulla C., and (1999). Sight and Insight-Oon the Physiological Role of Nitricoxide in the Visual System. Trends Neurosci. 22 (3), 109–116. 10.1016/s0166-2236(98)01299-5 10199635

[B14] Djakovic Z. Djakovic I. Cesarec V. Madzarac G. Becejac T. Zukanovic G. (2016). Esophagogastric Anastomosis in Rats: Improved Healing by BPC 157 and L-Arginine, Aggravated by L-NAME. World J. Gastroenterol. 22 (41), 9127–9140. 10.3748/wjg.v22.i41.9127 27895400 PMC5107594

[B15] Drmic D. Kolenc D. Ilic S. Bauk L. Sever M. Sever Z. (2017). Celecoxibinduced Gastrointestinal, Liver and Brain Lesions in Rats, Counteraction by BPC 157 or L-Arginine, Aggravation by L-NAME. World J. Gastroenterol. 23 (29), 5304–5312. 10.3748/wjg.v23.i29.5304 28839430 PMC5550779

[B16] Drmic D. Samara M. Vidovic T. Malekinusic D. Antunovic M. Vrdoljak B. (2018). Counteraction of Perforated Cecum Lesions in Rats: Effects of pentadecapeptideBPC 157, L-NAME and L-Arginine. World J. Gastroenterol. 24 (48), 5462–5476. 10.3748/wjg.v24.i48.5462 30622376 PMC6319139

[B17] Duzel A. Vlainic J. Antunovic M. Malekinusic D. Vrdoljak B. Samara M. (2017). Stable Gastric Pentadecapeptide BPC 157 in the Treatment of Colitis and Ischemiaand Reperfusion in Rats: New Insights. World J. Gastroenterol. 23 (48), 8465–8488. 10.3748/wjg.v23.i48.8465 29358856 PMC5752708

[B18] Faghihi H. Mehdi Rajaei S. Mehrazin H. Golabdar S. Brooks D. E. (2020). Effect Oftopical 1% Tetracaine Hydrochloride on Intraocular Pressure in Ophthalmologically Normalnorses; a Pilot Study. J. Equine Vet. Sci. 95, 103296. 10.1016/j.jevs.2020.103296 33276925

[B19] Gjurasin M. Miklic P. Zupancic B. Perovic D. Zarkovic K. Brcic L. (2010). Peptide Therapy with Pentadecapeptide BPC 157 in Traumatic Nerve Injury. Regul. Pept. 160 (1-3), 33–41. 10.1016/j.regpep.2009.11.005 19903499

[B20] Gojkovic S. Krezic I. Vrdoljak B. Malekinusic D. Barisic I. Petrovic A. (2020). Pentadecapeptide BPC 157 Resolves Suprahepatic Occlusion of the Inferior Cavalvein, Budd-Chiari Syndrome Model in Rats. World J. Gastrointest. Pathophysiol 11 (1), 1–19. 10.4291/wjgp.v11.i1.1 32226643 PMC7093306

[B21] Guthrie M. J. Kang-Mieler J. J. (2014). Dual Electroretinogram/nitric Oxide Carbon Fibermicroelectrode for Direct Measurement of Nitric Oxide in the *In Vivo* Retina. IEEE Transbiomed Eng. 61 (3), 611–619. 10.1109/TBME.2013.2281541 24043366

[B22] Gwyer D. Wragg N. M. Wilson S. L. (2019). Gastric Pentadecapeptide Body Protectioncompound BPC 157 and its Role in Accelerating Musculoskeletal Soft Tissue Healing. Celltissue Res. 377, 153–159. 10.1007/s00441-019-03016-8 30915550

[B23] Hangai M. Yoshimura N. Hiroi K. Mandai M. Honda Y. (1999). Role of Nitric Oxideduring the Initial Phase of Reperfusion after Retinal Ischemia in the Rat. Ophthalmic Res. 31 (1), 16–23. 10.1159/000055508 9831818

[B24] Hayreh S. S. van Heuven W. A. Hayreh M. S. (1978). Experimental Retinal Vascularocclusion. I. Pathogenesis of central Retinal Vein Occlusion. Arch. Ophthalmol. 96 (2), 311–323. 10.1001/archopht.1978.03910050179015 415709

[B25] Hrelec M. Klicek R. Brcic L. Brcic I. Cvjetko I. Seiwerth S. (2009). Abdominal Aorta Anastomosis in Rats and Stable Gastric Pentadecapeptide BPC 157, Prophylaxis and Therapy. J. Physiol. Pharmacol. 60 (7), 161–165. 20388960

[B26] Hsieh M. J. Lee C. H. Chueh H. Y. Chang G. J. Huang H. Y. Lin Y. (1997). Protective Effect of Nitric Oxide on Ischemic Retina. Nippon Ganka Gakkai Zasshi 101 (8), 639–643. 9284618

[B27] Hsieh M. J. Liu H. T. Wang C. N. Huang H. Y. Lin Y. Ko Y. S. (2017). Therapeutic Potential of Pro-angiogenic BPC157 Is Associated with VEGFR2 Activationand Up-Regulation. J. Mol. Med. 95 (3), 323–333. 10.1007/s00109-016-1488-y 27847966

[B28] Ilic S. Brcic I. Mester M. Filipovic M. Sever M. Klicek R. (2009). Over-dose Insulin and Stable Gastric Pentadecapeptide BPC 157. Attenuated Gastric Ulcers, Seizures, Brain Lesions, Hepatomegaly, Fatty Liver, Breakdown of Liver Glycogen, Profound Hypoglycemia and Calcification in Rats. J. Physiol. Pharmacol. 60 (7), 107–114. 20388953

[B29] Ilic S. Drmic D. Franjic S. Kolenc D. Coric M. Brcic L. (2011a). Pentadecapeptide BPC 157 and its Effects on a NSAID Toxicity Model: Diclofenacinducedgastrointestinal, Liver, and Encephalopathy Lesions. Life Sci. 88 (11-12), 535–542. 10.1016/j.lfs.2011.01.015 21295044

[B30] Ilic S. Drmic D. Zarkovic K. Kolenc D. Brcic L. Radic B. (2011b). Ibuprofenhepatic Encephalopathy, Hepatomegaly, Gastric Lesion and Gastric Pentadecapeptide BPC157 in Rats. Eur. J. Pharmacol. 667 (1-3), 322–329. 10.1016/j.ejphar.2011.05.038 21645505

[B31] Ilic S. Drmic D. Zarkovic K. Kolenc D. Coric M. Brcic L. (2010). Highhepatotoxic Dose of Paracetamol Produces Generalized Convulsions and Brain Damage Inrats. A Counteraction with the Stable Gastric Pentadecapeptide BPC 157 (PL 14736). Jphysiol Pharmacol. 61 (2), 241–250. 20436226

[B32] Ju W. K. Kim K. Y. Park S. J. Park D. K. Park C. B. Oh S. J. (2000). Nitricoxide Is Involved in Sustained and Delayed Cell Death of Rat Retina Following Transientischemia. Brain Res. 881 (2), 231–236. 10.1016/s0006-8993(00)02816-x 11036166

[B33] Kang E. A. Han Y. M. An J. M. Park Y. J. Sikiric P. Kim D. H. (2018). BPC157as Potential Agent Rescuing from Cancer Cachexia. Curr. Pharm. Des. 24 (18), 1947–1956. 10.2174/1381612824666180614082950 29898649

[B34] Klicek R. Kolenc D. Suran J. Drmic D. Brcic L. Aralica G. (2013). Stablegastric Pentadecapeptide BPC 157 Heals Cysteamine-Colitis and colon-colon-anastomosisand Counteracts Cuprizone Brain Injuries and Motor Disability. J. Physiol. Pharmacol. 64 (5), 597–612. 24304574

[B35] Kokot A. Zlatar M. Stupnisek M. Drmic D. Radic R. Vcev A. (2016). NOsystem Dependence of Atropine-Induced Mydriasis and L-NAME- and L-Arginine-Inducedmiosis: Reversal by the Pentadecapeptide BPC 157 in Rats and guinea Pigs. Eur. Jpharmacol 771, 211–219. 10.1016/j.ejphar.2015.12.016 26698393

[B36] Kolovrat M. Gojkovic S. Krezic I. Malekinusic D. Vrdoljak B. Kasnik Kovac K. (2020). Pentadecapeptide BPC 157 Resolves Pringle Maneuver in Rats, Both Ischemia Andreperfusion. World J. Hepatol. 12 (5), 184–206. 10.4254/wjh.v12.i5.184 32547687 PMC7280862

[B37] Konosic S. Petricevic M. Ivancan V. Konosic L. Goluza E. Krtalic B. (2019). Intragastric Application of Aspirin, Clopidogrel, Cilostazol, and BPC 157 in Rats: Platelet Aggregation and Blood Clot. Oxid Med. Cel Longev, 9084643. 10.1155/2019/9084643 PMC6955135 31976029

[B38] Kovalcuka L. Birgele E. Bandere D. Williams D. L. (2013). The Effects of Ketaminehydrochloride and Diazepam on the Intraocular Pressure and Pupil Diameter of the Dog's Eye. Vet. Ophthalmol. 16 (1), 29–34. 10.1111/j.1463-5224.2012.01015.x 23294621

[B39] Lazic R. Gabric N. Dekaris I. Bosnar D. Boban-Blagaic A. Sikiric P. (2005). Gastric Pentadecapeptide BPC 157 Promotes Corneal Epithelial Defects Healing in Rats. Coll. Antropol 29 (1), 321–325. 16117343

[B40] Lojo N. Rasic Z. Zenko Sever A. Kolenc D. Vukusic D. Drmic D. (2016). Effects of Diclofenac, L-NAME, L-Arginine, and Pentadecapeptide BPC 157 Ongastrointestinal, Liver, and Brain Lesions, Failed Anastomosis, and Intestinal Adaptationdeterioration in 24 hour-Short-Bowel Rats. PLoS One 11 (9), e0162590. 10.1371/journal.pone.0162590 27627764 PMC5023193

[B41] Luetic K. Sucic M. Vlainic J. Halle Z. B. Strinic D. Vidovic T. (2017). Cyclophosphamide Induced Stomach and Duodenal Lesions as a NO-System Disturbance Inrats: L-NAME, L-Arginine, Stable Gastric Pentadecapeptide BPC 157. nflammopharmacology 25 (2), 255–264. 10.1007/s10787-017-0330-7 28255738

[B42] Masnec S. Kokot A. Zlatar M. Kalauz M. Kunjko K. Radic B. (2015). Perforating Corneal Injury in Rat and Pentadecapeptide BPC 157. Exp. Eye Res. 136, 9–15. 10.1016/j.exer.2015.04.016 25912999

[B43] Medvidovic-Grubisic M. Stambolija V. Kolenc D. Katancic J. Murselovic T. Plestina-Borjan I. (2017). Hypermagnesemia Disturbances in Rats, NO-Related: Pentadecapeptide BPC 157 Abrogates, L-NAME and L-Arginine Worsen. Inflammopharmacology 25 (4), 439–449. 10.1007/s10787-017-0323-6 28210905

[B44] Mermoud A. Baerveldt G. Minckler D. S. Lee M. B. Rao N. A. (1994). Intraocularpressure in Lewis Rats. Invest. Ophthalmol. Vis. Sci. 35 (5), 2455–2460. 8163335

[B45] Minhas G. Morishita R. Shimamura M. Bansal R. Anand A. (2015). Modeling Transient Retinal Ischemia in Mouse by Ligation of Pterygopalatine Artery. Ann. Neurosci. 22 (4), 222–225. 10.5214/ans.0972.7531.220406 26526209 PMC4627200

[B46] Nagaoka T. Takashi S. Fumihiko M. Eiichi S. Akitoshi Y. (2002). The Effect of Nitricoxide on Retinal Blood Flow during Hypoxia in Cats. Invest. Ophthalmol. Vis. Sci. 43 (9), 3037–3044. 12202527

[B47] Osborne N. N. Casson R. J. Wood J. P. Chidlow G. Graham M. Melena J. (2004). Retinal Ischemia: Mechanisms of Damage and Potential Therapeutic Strategies. Prog. Retineye Res. 23 (1), 91–147. 10.1016/j.preteyeres.2003.12.001 14766318

[B48] Ostwald P. Goldstein I. M. Pachnanda A. Roth S. (1995). Effect of Nitric Oxide Synthaseinhibition on Blood Flow after Retinal Ischemia in Cats. Invest. Ophthalmol. Vis. Sci. 36 (12), 2396–2403. 7591629

[B49] Ostwald P. Park S. S. Toledano A. Y. Roth S. (1997). Adenosine Receptor Blockadeand Nitric Oxide Synthase Inhibition in the Retina: Impact upon post-ischemic Hyperemiaand. the Electroretinogram. Vis. Res. 37 (24), 3453–3461. 10.1016/S0042-6989(96)00222-2 9425522

[B50] Park J. M. Lee H. J. Sikiric P. Hahm K. B. (2020). BPC 157 Rescued NSAIDcytotixicityvia Stabilizing Intestinal Permeability and Enhancing Cytoprotection. CurrPharm Des 26 (25), 2971–2981. 10.2174/1381612826666200523180301 32445447

[B51] Pereiro X. Ruzafa N. Urcola J. H. Sharma S. C. Vecino E. (2020). Differentialdistribution of RBPMS in Pig, Rat and Human Retina after Damage. Int. J. Mol. Sci. 21 (23), 9330. 10.3390/ijms21239330 PMC7729751 33297577

[B52] Perovic D. Kolenc D. Bilic V. Somun N. Drmic D. Elabjer E. (2019). Stablegastric Pentadecapeptide BPC 157 Can Improve the Healing Course of Spinal Cord Injuryand lead to Functional Recovery in Rats. J. Orthop. Surg. Res. 14 (1), 199. 10.1186/s13018-019-1242-6 31266512 PMC6604284

[B53] Portney G. L. Purcell T. W. (1975). The Influence of Tropicamide on Intraocular Pressure. AnnOphthalmol 7 (1), 31–34. 1111414

[B54] Ruenzi M. Stolte M. Veljaca M. Oreskovic K. Peterson J. (2005). A Multicenter, Randomized, Double Blind, Placebo Controlled Phase II Study of PL 14736 Enema in the Treatment of Mild-To-Moderate Ulcerative Colitis, Ulcerative Colitis Study Group. Gastroenterology 128, A584.

[B55] Sakamoto K. Yonoki Y. Kubota Y. Kuwagata M. Saito M. Nakahara T. (2006). Inducible Nitric Oxide Synthase Inhibitors Abolished Histological protection by Lateischemic Preconditioning in Rat Retina. Exp. Eye Res. 82 (3), 512–518. 10.1016/j.exer.2005.08.011 16198335

[B56] Sarchahi A. A. Bozorgi H. (2012). Effect of Tetracaine on Intraocular Pressure in normal Andhypertensive Rabbit Eyes. J. Ophthalmic Vis. Res. 7 (1), 29–33. 22737384 PMC3381104

[B57] Seiwerth S. Rucman R. Turkovic B. Sever M. Klicek R. Radic B. (2018). BPCstandard Angiogenic Growth Factors. Gastrointestinal Tract Healing, Lessons Fromtendon, Ligament, Muscle and Bone Healing. Curr. Pharm. Des. 24 (18), 1972–1989. 10.2174/1381612824666180712110447 29998800

[B58] Sever A. Z. Sever M. Vidovic. T. Lojo N. Kolenc D. Vuletic L. B. (2019). Stable Gastric Pentadecapeptide BPC 157 in the Therapy of the Rats with Bile Duct Ligation. Eur. J. Pharmacol. 847, 130–142. 10.1016/j.ejphar.2019.01.030 30690000

[B59] Sikiric P. Hahm K. B. Blagaic A. B. Tvrdeic A. Pavlov K. H. Petrovic A. (2020). Table Gastric Pentadecapeptide BPC 157, Robert’s Stomachcytoprotection/adaptive Cytoprotection/organoprotection, and Selye’s Stress Copingresponse: Progress, Achievements, and the Future, Gut Liver, 14, 153–167. 10.5009/gnl18490 31158953 PMC7096228

[B60] Sikiric P. Rucman R. Turkovic B. Sever M. Klicek R. Radic B. (2018). Novel Cytoprotective Mediator, Stable Gastric Pentadecapeptide BPC 157. Vascularrecruitment and Gastrointestinal Tract Healing. Curr. Pharm. Des. 24 (18), 1990–2001. 10.2174/1381612824666180608101119 29879879

[B61] Sikiric P. Seiwerth S. Grabarevic Z. Rucman R. Petek M. Jagic V. (1997). The Influence of a Novel Pentadecapeptide, BPC 157, on N(G)-nitro-L-argininemethylester and L-Arginine Effects on Stomach Mucosa Integrity and Blood Pressure. Eur. Jpharmacol 332 (1), 23–33. 10.1016/s0014-2999(97)01033-9 9298922

[B62] Sikiric P. Seiwerth S. Rucman R. Kolenc D. Vuletic L. B. Drmic D. (2016). Brain-gut axis and Pentadecapeptide BPC 157: Theoretical and Practical Implications. Curr. Neuropharmacol 14 (8), 857–865. 10.2174/1570159x13666160502153022 27138887 PMC5333585

[B63] Sikiric P. Seiwerth S. Rucman R. Turkovic B. Rokotov D. S. Brcic L. (2014). Stable Gastric Pentadecapeptide BPC 157-NO-System Relation. Curr. Pharm. Des. 20 (7), 1126–1135. 10.2174/13816128113190990411 23755725

[B64] Sikiric P. Seiwerth S. Rucman R. Turkovic B. Rokotov D. S. Brcic L. (2013). Toxicity by NSAIDs. Counteraction by Stable Gastric Pentadecapeptide BPC 157. CurrPharm Des 19 (1), 76–83. 10.2174/13816128130111 22950504

[B65] Stupnisek M. Franjic S. Drmic D. Hrelec M. Kolenc D. Radic B. (2012). Pentadecapeptide BPC 157 Reduces Bleeding Time and Thrombocytopenia after Amputationin Rats Treated with Heparin, Warfarin or Aspirin. Thromb. Res. 129 (5), 652–659. 10.1016/j.thromres.2011.07.035 21840572

[B66] Stupnisek M. Kokot A. Drmic D. Hrelec Patrlj M. Zenko Sever A. Kolenc D. (2015). Pentadecapeptide BPC 157 Reduces Bleeding and Thrombocytopenia Afteramputation in Rats Treated with Heparin, Warfarin, L-NAME and L-Arginine. PLoS One 10 (4), e0123454. 10.1371/journal.pone.0123454 25897838 PMC4405609

[B67] Sucic M. Luetic K. Jandric I. Drmic D. Sever A. Z. Vuletic L. B. (2019). Therapy of the Rat Hemorrhagic Cystitis Induced by Cyclophosphamide. Stable Gastricpentadecapeptide BPC 157, L-Arginine, L-NAME. Eur. J. Pharmacol. 861, 172593. 10.1016/j.ejphar.2019.172593 31401154

[B68] Tkalcevic V. I. Cuzic S. Brajsa K. Mildner B. Bokulic A. Situm K. (2007). Enhancement by PL 14736 of Granulation and Collagen Organization in Healing Woundsand the Potential Role of Egr-1 Expression. Eur. J. Pharmacol. 570 (1-3), 212–221. 10.1016/j.ejphar.2007.05.072 17628536

[B69] Toda N. Nakanishitoda M. (2007). Nitric Oxide: Ocular Blood Flow, Glaucoma, Anddiabetic Retinopathy. Prog. Retin. Eye Res. 26 (3), 205–238. 10.1016/j.preteyeres.2007.01.004 17337232

[B70] Tohyama Y. Sikiric P. Diksic M. (2004). Effects of Pentadecapeptide BPC157 Onregional Serotonin Synthesis in the Rat Brain: α-Methyl-L-tryptophan Autoradiographicmeasurements. Life Sci. 76 (3), 345–357. 10.1016/j.lfs.2004.08.010 15531385

[B71] Tudor M. Jandric I. Marovic A. Gjurasin M. Perovic D. Radic B. (2010). Traumatic Brain Injury in Mice and Pentadecapeptide BPC 157 Effect. Regul. Pept. 160 (1- 3), 26–32. 10.1016/j.regpep.2009.11.012 19931318

[B72] Turkovic B. Sikiric P. Seiwerth S. Mise S. Anic T. Petek M. (2004). Stablegastric Pentadecapeptide BPC 157 Studied for Inflammatory Bowel Disease (PLD-116, PL14736, Pliva) Induces Nitric Oxide Synthesis. Gastroenterology 126, 287.

[B73] Veljaca M. Pavic-Sladojev D. Mildner B. Brajsa K. Krnic Z. Bibenik M. (2003). Safety, Tolerability and Pharmacokinetics of PL 14736, a Novel Agent for Treatment of Ulcerative Colitis, in Healthy Male Volunteers. Gut 51 (Suppl. III), A309.

[B74] Vestergaard N. Cehofski L. J. Honoré B. Aasbjerg K. Vorum H. (2019). Animal Models Used to Stimulate Retinal Artery Occlusion: A Comprehensive Review. Transl Vis. Sci. Technol. 8 (4), 23. 10.1167/tvst.8.4.23 PMC6701503 31440422

[B75] Vukojevic J. Siroglavic M. Kasnik K. Kralj T. Stancic D. Kokot A. (2018). Rat Inferior Caval Vein (ICV) Ligature and Particular New Insights with the Stable Gastricpentadecapeptide BPC 157. Vascul Pharmacol. 106, 54–66. 10.1016/j.vph.2018.02.010 29510201

[B76] Vukojevic J. Vrdoljak B. Malekinusic D. Siroglavic M. Milavic M. Kolenc D. (2020). The Effect of Pentadecapeptide BPC 157 on Hippocampal Ischemia/reperfusioninjuries in Rats. Brain Behav. 10 (8), e01726. 10.1002/brb3.1726 32558293 PMC7428500

[B77] Wang X. Y. Qu M. Duan R. Shi D. Jin L. Gao J. (2019). Cytoprotectivemechanism of the Novel Gastric Peptide BPC157 in Gastrointestinal Tract and Culturedenteric Neurons and Glial Cells. Neurosci. Bull. 35 (1), 167–170. 10.1007/s12264-018-0269-8 30116973 PMC6357276

[B78] Wu H. Wei M. Li N. Lu Q. Shrestha S. M. Tan J. (2020). Clopidogrel-induced Gastric Injury in Rats Is Attenuated by Stable Gastric Pentadecapeptide BPC 157. Drug Des. Devel Ther. 14, 5599–5610. 10.2147/DDDT.S284163 PMC7763470 33376304

[B79] Xu C. Sun L. Ren F. Huang P. Tian Z. Cui J. (2020). Preclinical Safety Evaluation of Body Protective Compound-157, a Potential Drug for Treating Various Wounds. Regul. Toxicol. Pharmacol. 114, 104665. 10.1016/j.yrtph.2020.104665 32334036

[B80] Zemba Cilic A. Zemba M. Cilic M. Balenovic I. Strbe S. Ilic S. (2021). Pentadecapeptide BPC 157 Counteracts L-NAME-Induced Catalepsy. BPC 157, L-NAME, L-Arginine, NO-Relation the Suited Rat Acute and Chronic Models Resembling 'positive-Like'symptoms of Schizophrenia. Behav. Brain Res. 396, 112919. 10.1016/j.bbr.2020.112919 32956773

